# Hydrogen sulfide dysregulates the immune response by suppressing central carbon metabolism to promote tuberculosis

**DOI:** 10.1073/pnas.1919211117

**Published:** 2020-03-05

**Authors:** Md. Aejazur Rahman, Bridgette M. Cumming, Kelvin W. Addicott, Hayden T. Pacl, Shannon L. Russell, Kievershen Nargan, Threnesan Naidoo, Pratistadevi K. Ramdial, John H. Adamson, Rui Wang, Adrie J. C. Steyn

**Affiliations:** ^a^Africa Health Research Institute, 4001 Durban, KwaZulu Natal, South Africa;; ^b^Department of Microbiology, University of Alabama at Birmingham, Birmingham, AL 35294;; ^c^Department of Anatomical Pathology, National Health Laboratory Service, Inkosi Albert Luthuli Central Hospital, University of KwaZulu-Natal, 4091 Durban, South Africa;; ^d^Department of Biology, York University, Toronto, ON M3J 1P3, Canada;; ^e^Centers for AIDS Research and Free Radical Biology, University of Alabama at Birmingham, Birmingham, AL 35294

**Keywords:** tuberculosis, H_2_S, hydrogen sulfide, pathogenesis, metabolism

## Abstract

The ubiquitous gasotransmitter hydrogen sulfide (H_2_S) has been recognized to play a crucial role in human health. Using cystathionine γ-lyase (CSE)-deficient mice, we demonstrate an unexpected role of H_2_S in *Mycobacterium tuberculosis* (*Mtb*) pathogenesis. We showed that *Mtb-*infected CSE^−/−^ mice survive longer than WT mice, and support reduced pathology and lower bacterial burdens in the lung, spleen, and liver. Similarly, in vitro *Mtb* infection of macrophages resulted in reduced colony forming units in CSE^−/−^ cells. Chemical complementation of infected WT and CSE^−/−^ macrophages using the slow H_2_S releaser GYY3147 and the CSE inhibitor DL-propargylglycine demonstrated that H_2_S is the effector molecule regulating *Mtb* survival in macrophages. Furthermore, we demonstrate that CSE promotes an excessive innate immune response, suppresses the adaptive immune response, and reduces circulating IL-1β, IL-6, TNF-α, and IFN-γ levels in response to *Mtb* infection. Notably, *Mtb* infected CSE^−/−^ macrophages show increased flux through glycolysis and the pentose phosphate pathway, thereby establishing a critical link between H_2_S and central metabolism. Our data suggest that excessive H_2_S produced by the infected WT mice reduce HIF-1α levels, thereby suppressing glycolysis and production of IL-1β, IL-6, and IL-12, and increasing bacterial burden. Clinical relevance was demonstrated by the spatial distribution of H_2_S-producing enzymes in human necrotic, nonnecrotic, and cavitary pulmonary tuberculosis (TB) lesions. In summary, CSE exacerbates TB pathogenesis by altering immunometabolism in mice and inhibiting CSE or modulating glycolysis are potential targets for host-directed TB control.

## Significance

Tuberculosis (TB) is responsible for millions of deaths each year and several billion people are latently infected with *Mycobacterium tuberculosis* (*Mtb*). *Mtb* modulates host factors, such as endogenous gaseous signalling molecules, to persist in humans for decades. H_2_S has diverse biological functions, including modulation of immunity and cellular respiration. However, the role of H_2_S in TB is unclear. We found that mice deficient in H_2_S production are more resistant to *Mtb* infection than WT mice. Upon infection, *Mtb* increases host H_2_S, which suppresses central carbon metabolism and increases inflammation. Distribution of H_2_S-producing enzymes in human TB lungs showed that H_2_S is produced at the site of infection. These findings identify glycolysis and H_2_S-producing enzymes as targets for TB host-directed therapies.

Tuberculosis (TB) is a widespread infectious disease of humans, caused by *Mycobacterium tuberculosis* (*Mtb*). Endogenous gaseous signaling molecules, such as nitric oxide (NO) and carbon monoxide (CO), produced by inducible nitric oxide synthase (iNOS) and heme oxygenase-1 (HO-1), respectively, play important roles in innate immunity against mycobacterial disease progression (1, 2). Recently, a third gasotransmitter, hydrogen sulfide (H_2_S), has received much attention because of its crucial role in numerous pathophysiological conditions, paving the way for innovative therapeutic intervention strategies (3–5). Among the gasotransmitters, H_2_S is the most chemically reactive, technically the most difficult to work with, and has biologically diverse functions that profoundly affect most organ systems in humans and animals (4). In mammals, H_2_S is primarily synthesized by two enzymes responsible for metabolism of l-cysteine (Cys), cystathionine β-synthase (CBS) and cystathionine γ-lyase (CSE), and a third pathway that involves the combined action of 3-mercaptopyruvate sulfurtransferase (MPST) and cysteine aminotransferase (5, 6). The biochemical activity of H_2_S is highly divergent (7); it rapidly travels through cell membranes without transporters and critically depends on its local concentrations and enzymatic production (8). Low concentrations of H_2_S stimulate mitochondrial oxidative phosphorylation (OXPHOS), cellular bioenergetics, and show antiinflammatory effects (9, 10). However, supraphysiological concentrations of H_2_S have adverse effects, including inhibition of OXPHOS, stimulation of pro-oxidant and proinflammatory effects, and promoting cellular necrosis and apoptosis (9, 10).

Histopathological analyses of human TB lung tissue have shown that overwhelming inflammation triggers severe immunopathology, which is associated with excessive neutrophil recruitment (11). The H_2_S signaling pathway is associated with numerous inflammatory diseases including, but not limited to, rheumatoid arthritis (12), burn injury (13), acute pancreatitis (14), and septic shock (15). During septic shock, H_2_S synthesizing activity, neutrophil infiltration, and proinflammatory cytokine levels increase significantly (13–15). Notably, CSE-deletion mice had significantly reduced inflammation following sepsis (16), suggesting that CSE exacerbates inflammation in this model system. H_2_S is also an endogenous potentiator of T cell activation (17), which is essential for the control of *Mtb* infection.

Metabolism plays an important role in the regulation of immunity. Notably, LPS- and IFN-γ–activated inflammatory macrophages have enhanced glycolysis and impaired OXPHOS (18). Recent literature reported how glycolytic enzymes support proinflammatory macrophage functions (19). In particular, pyruvate kinase M2 forms a complex with hypoxia-inducible factor-1α (HIF-1α) to promote IL-1β expression and it also phosphorylates STAT3 to boost IL-6 and IL-1β expression (20). More recently, we have demonstrated that *Mtb* infection of human monocyte-derived macrophages depresses both glycolysis and OXPHOS of the infected macrophage (21) and that *Mtb* infection leads to a progressive decline in metabolic health of effector T cells (22), suggesting that *Mtb* rewires host immunometabolism to establish disease.

Surprisingly, despite many vital physiological and overlapping functions with NO and CO, the role of host H_2_S in bacterial pathogenesis, and TB in particular, is unclear and represents a gap in the field. Hence, establishing how host-generated H_2_S regulates the immunometabolism of TB is important as it may help identify new host-directed therapeutic targets, and contribute to a broader understanding of how gasotransmitters can be engineered as an approach to therapy. In this study, we hypothesize that CSE-generated H_2_S regulates bacillary burden by altering host immunometabolism. This hypothesis is based on the widely known role of H_2_S as a gasotransmitter in regulating cellular energy metabolism (23) and inflammation (24). To test this hypothesis, we examined the cellular and spatial distribution of CSE, CBS, and MPST within the microenvironment of resected human TB lungs, and we used CSE^−/−^ mice as a model system for *Mtb* infection studies (25). We examined the immune cell distribution in mouse lungs and the mouse serum cytokine levels. We also measured H_2_S levels during macrophage infection and determined cytokine levels secreted by chemically complemented CSE^−/−^ macrophages. Finally, we used real-time extracellular flux analysis and liquid chromatography/mass spectrometry (LC-MS/MS) to examine the role of CSE in central energy metabolism.

## Results

### Cellular and Lesional Distribution of CSE, CBS, and MPST in Human TB Lungs.

**Fig. 1.**
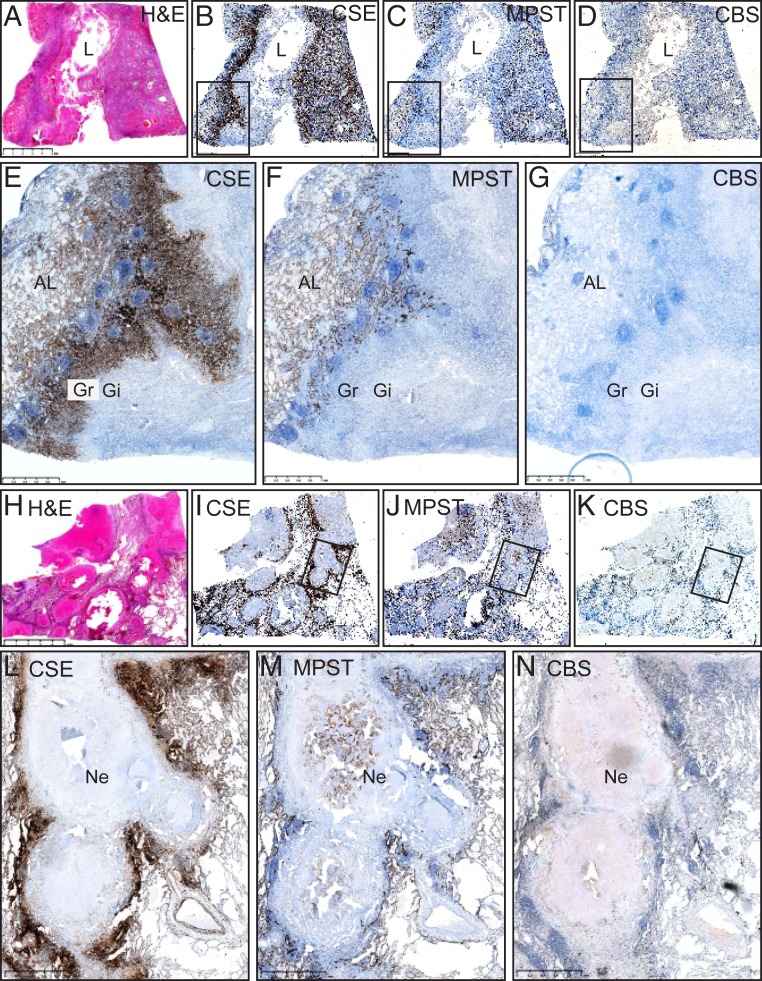
Spatial distribution of CSE, CBS, and MPST in the human TB lung. Low power hematoxylin and eosin (H&E) demonstration of a TB cavity (*A*) and IHC staining of CSE (*B*), MPST (*C*), and CBS (*D*). Medium power depiction of IHC staining of CSE (*E*), MPST (*F*), and CBS (*G*). Low power H&E demonstration of caseous necrotic granuloma (*H*) and IHC staining of CSE (*I*), MPST (*J*), and CBS (*K*). Medium power depiction of IHC staining of CSE (*L*), MPST (*M*), and CBS (*N*). AL, adjacent lung; Gi, granulomatous inflammation layer; Gr, granulation layer; L, lumen; Ne, necrosis. (Scale bars: *A–D*, 5 mm as presented in *A*; *E–G*, 1 mm; *H–K*, 5 mm as presented in *H*; *L–N*, 1 mm.)

Excessive H_2_S levels dysregulate cellular homeostasis and are associated with maladaptive inflammation and cell death (23, 24). Hence, it is important to examine the cellular and lesional distribution of CSE, CBS, and MPST in human tuberculous lung tissue, as it will establish their clinical relevance. Here, we appraise pathological features of necrotizing lung sections, including cavitary TB and tubercle formation in two human test cases and control lung sections. Test case 1 demonstrates sections of a lung with a TB cavity wall and adjacent lung tissue (Fig. 1 *A*–*G*). Noticeably, TB lung tissues were virtually unstained by CBS antibodies in both cases and only a few isolated cells stained weakly positive for CBS (Fig. 1 *D*, *G*, *K*, and *N* and *SI Appendix*, Fig. S1*C*). The TB cavity wall, including the granulomatous layer and adluminal necrotic components, was unstained by CSE (Fig. 1 *B* and *E* and *SI Appendix*, Fig. S1*A*) and MPST antibodies (Fig. 1 *C* and *F* and *SI Appendix*, Fig. S1*B*). Myofibroblasts and histiocytes in the cavity wall (Fig. 1 *B* and *E* and *SI Appendix*, Fig. S1*A*) and the vascular mural smooth muscle (*SI Appendix*, Fig. S2*A*) stained strongly positive for CSE. The alveolar pneumocytes (Fig. 1 *C* and *F* and *SI Appendix*, Figs. S1*B* and S3 *B* and *D*), the bronchiolar epithelium (*SI Appendix*, Fig. S2 *B* and *E*), and the adjacent lung were brightly stained for MPST, whereas the bronchiolar epithelium was stained substantially weaker for CSE (*SI Appendix*, Fig. S2 *A* and *D*). CSE and MPST compartmentalize to the cytosol, or nucleus and cytosol (*SI Appendix*, Fig. S3).

Test case 2 demonstrated sections of tubercles at varying stages of development and inflammatory organization (Fig. 1 *H*–*N*). High magnification revealed that the granulomatous layer and central necrotic component of some tubercles were completely unstained by CSE (*SI Appendix*, Fig. S4*A*) and MPST antibodies (*SI Appendix*, Fig. S4*B*). Curiously, some tubercles demonstrated an organoid MPST staining pattern in the central necrotic component, compatible with ghost outlines of alveolar spaces and septa (*SI Appendix*, Fig. S4*C*) and is indicative of an early stage of necrosis of MPST^+^ cells. This raises the possibility that H_2_S could function as a cellular fuel source under hypoxic conditions since these enzymes (MPST, CSE, and CBS) do not require oxygen as a cofactor. Histiocytes and giant cells stain positive for CSE antibodies (*SI Appendix*, Fig. S5). CSE and MPST demonstrated similar staining of the adjacent lung as in test case 1.

Positive control human liver sections demonstrated intense CSE, CBS, and MPST staining (*SI Appendix*, Fig. S6). Control sections of a healthy human lung with normal alveolar spaces, septa, vascular, and bronchiolar components demonstrated more intense CSE (*SI Appendix*, Fig. S7*A*) than MPST staining (*SI Appendix*, Fig. S7*B*) of alveolar pneumocytes, respiratory and terminal bronchiolar epithelium, circulating monocytes, scattered desquamated epithelial cells, and vascular smooth muscle. In contrast, CBS stained negative in these healthy lung tissues (*SI Appendix*, Fig. S7*C*). Negative controls using secondary antibody alone or isotope control antibody demonstrated immune negative reactions, confirming the specificity of CSE, CBS, and MPST staining (*SI Appendix*, Fig. S7*D*).

In sum, a histopathological appraisal of human TB lung cavitary and TB lesions demonstrated the spatial distribution of CSE and MPST and lack of CBS within the lung TB microenvironment. The distinct architectural and cellular patterned responses were reflected by giant cells, histiocytes, fibroblasts, epithelial and smooth muscle cells, and alveolar pneumocytes that stained positive for the H_2_S-producing enzymes. Overall, compared to the healthy lung tissue, there is a marked increase in H_2_S-producing cells around cavitary and necrotic lesions, suggesting that *Mtb* pathogenesis triggers excessive H_2_S production. These findings provide key evidence for the clinical significance of H_2_S-producing enzymes in the pathophysiology of human pulmonary TB.

### CSE Exacerbates TB Disease in the Murine Model.

**Fig. 2.**
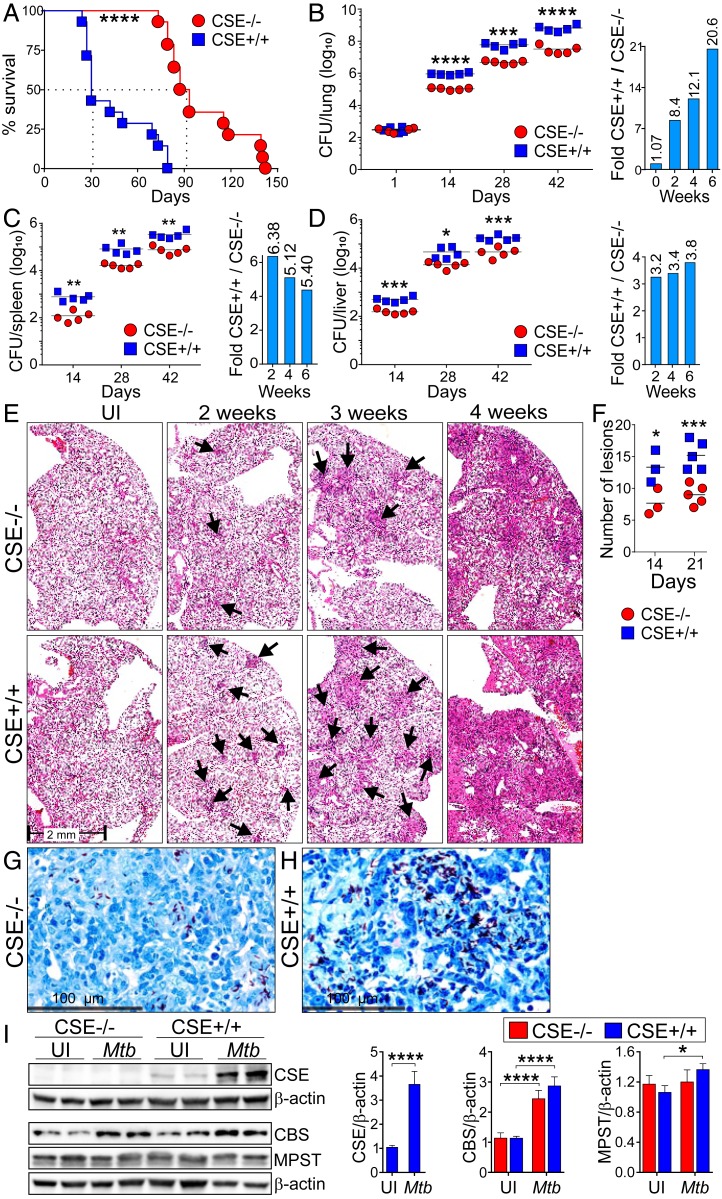
CSE promotes *Mtb* growth in mice. CSE^−/−^ and WT (CSE^+/+^) mice were infected with *Mtb* and observed for the degree of disease severity. (*A*) Survival study of *Mtb*-infected CSE^−/−^ and WT mice (*n* = 14). Bacterial burden (CFU) in the (*B*) lung, (*C*) spleen, and (*D*) liver of *Mtb*-infected mice over the course of infection. Bar graphs in the *Right* panels represent the fold-change in CFU between WT and CSE^−/−^ mice (*n* = 5). (*E*) Representative images of hematoxylin and eosin (H&E)-stained mouse lung sections of *Mtb*-infected mice over the course of infection. Arrows point to lesions. (*F*) Number of granulomatous lesions observed in the mouse lung sections stained with H&E after 14 and 21 d postinfection. Each symbol represents a mouse. Representative images of Ziehl–Neelsen stained (ZN) bacilli in *Mtb*-infected (*G*) CSE^−/−^ and (*H*) WT mouse lung sections on day 21 postinfection. (Scale bar, 100 µm.) (*I*) Western blot showing production of CSE, CBS, and MPST in lungs of uninfected (UI) and *Mtb*-infected CSE^−/−^ and WT mice at 3 wk postinfection (*n* = 2). Densitometric analysis of the Western blot bands are shown as relative protein expression normalized to β-actin band intensities. The relative protein expression observed in the uninfected WT mice has been normalized to 1. The unpaired Student *t* test was used for colony forming units (CFU) data and the two-way ANOVA was used for all other data. Data are representative of two independent experiments; ****P < 0.0001; ***P < 0.001; **P < 0.01; *P < 0.05.

To investigate the role of CSE in the pathogenesis of TB, homozygous CSE^−/−^ and WT mice were infected with *Mtb* and the pathology and organ burden were examined at different time points postinfection. We confirmed the deletion of the *CSE* allele in CSE^−/−^ mice via genotyping (*SI Appendix*, Fig. S8). Intriguingly, we discovered that CSE exacerbates TB. For example, the median survival for the infected CSE^−/−^ mice was 91 d compared to the 31 d of the infected WT mice (Fig. 2*A*). Although the CSE^−/−^ strain was derived from a C57BL/6J × 129SvEv background, of which the latter was shown to be significantly more susceptible to pneumococcal infection than C57BL/6J mice (26), we observed no major histological differences between the uninfected WT and CSE^−/−^ lungs that might have contributed to the significant survival of the infected CSE^−/−^ mice (*SI Appendix*, Fig. S9). Furthermore, we noticed increased karyorrhexis in the WT lungs, which was not observed in the CSE^−/−^ lungs at week 3 postinfection (*SI Appendix*, Fig. S10), and likely contributes toward the reduced survival of WT mice. At 2, 4, and 6 wk postinfection, the *Mtb* burden in the lungs of the CSE^−/−^ mice was significantly lower than in the WT mice (Fig. 2*B*). Furthermore, the bacillary burden in the spleen (Fig. 2*C*) and liver (Fig. 2*D*) was significantly higher in the WT mice than that in the CSE^−/−^ mice after 2, 4, and 6 wk of infection. A histopathological appraisal showed increased consolidated tissue in the lungs of *Mtb*-infected WT mice over time (Fig. 2*E*), and the number of granulomatous lesions in the WT mouse lungs were higher than in the CSE^−/−^ mice (Fig. 2*F*). Finally, microscopic analysis of acid-fast stained lung sections provides further evidence of higher bacillary loads in the infected WT mice than in the CSE^−/−^ mice (Fig. 2 *G* and *H*). Western blots of lung lysates confirmed substantial up-regulation of CSE in the lungs of infected WT mice (Fig. 2*I*). *Mtb* infection increased the expression of CBS in both WT and CSE^−/−^ lungs, but only modestly increased the expression of MPST in the WT mouse lungs (Fig. 2*I*). In sum, the survival duration, organ burden, and pathology data provide strong evidence that CSE exacerbates *Mtb* infection and disease.

### CSE Increases Myeloid Cells and Reduces Lymphoid Cells, and Decreases the Production of Cytokines That Control TB.

**Fig. 3.**
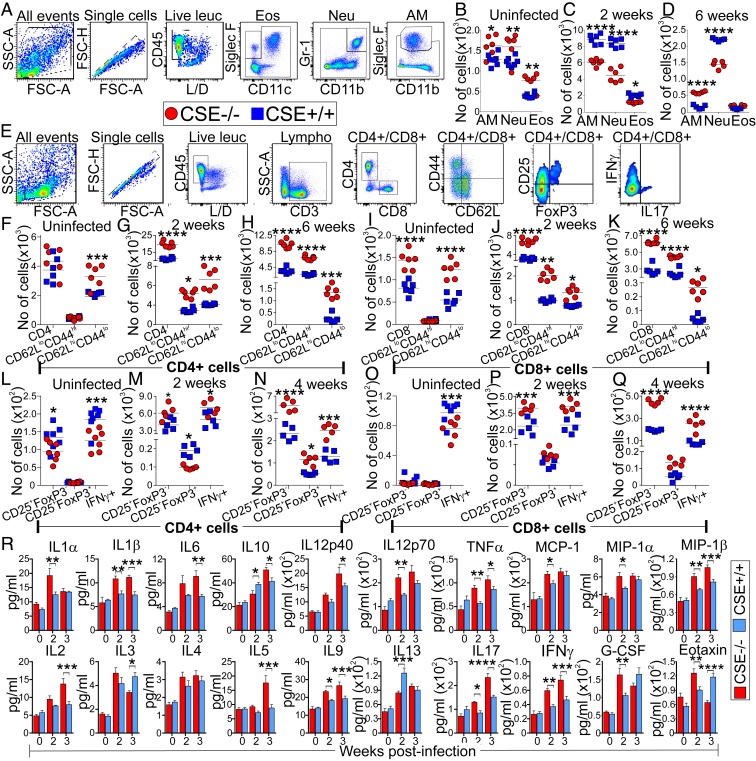
CSE regulates innate and adaptive immune responses in *Mtb*-infected mice. (*A*) Gating strategies used to characterize myeloid cells in the mouse lungs over the course of infection. Numbers of myeloid cell populations in the lungs of (*B*) uninfected mice, and at (*C*) 2 wk and (*D*) 6 wk postinfection. AM, alveolar macrophages; Eos, eosinophils; Neu, neutrophils. (*E*) Gating strategies used to characterize T cell subsets. Numbers of CD4^+^ T cells and their effector memory (CD62L^lo^CD44^hi^) and naïve cells (CD62L^hi^CD44^lo^) in the lungs of (*F*) uninfected mice, and at (*G*) 2 wk and (*H*) 6 wk postinfection. Numbers of CD8^+^ T cells and their effector memory and naïve cells in lungs of (*I*) uninfected mice, and at (*J*) 2 wk and (*K*) 6 wk postinfection. Number of CD4^+^ Treg (CD25^+^FoxP3^−^ and CD25^+^FoxP3^+^) and CD4^+^IFN-γ^+^ cells in the lungs of (*L*) uninfected mice, and at (*M*) 2 wk and (*N*) 4 wk postinfection. Number of CD8^+^ Treg (CD25^+^FoxP3^−^ and CD25^+^FoxP3^+^) and CD8^+^IFN-γ^+^ cells in the lungs of (*O*) uninfected mice, and at (*P*) 2 wk and (*Q*) 4 wk postinfection. Each symbol represents a mouse; five to six mice were used per group per time point. Data are representative of two independent experiments. (*R*) Cytokine levels in the serum of uninfected mice and at 2 and 3 wk postinfection. Data are represented as the mean ± SEM of three to four mice. Two-way ANOVA was used to determine statistical significance; ****P < 0.0001; ***P < 0.001; **P < 0.01; *P < 0.05.

Having shown that CSE exacerbates TB, we examined the host immune responses to *Mtb* infection in CSE^−/−^ and WT mice using flow cytometry and the gating strategies depicted in Fig. 3 *A* and *E*. Furthermore, we measured the cytokines in the mouse serum. Overall, our data demonstrate that CSE contributes significantly to immune dysregulation, which is supported by several lines of evidence.

First, increased numbers of alveolar macrophages, neutrophils, and eosinophils were observed in the lungs of uninfected CSE^−/−^ mice compared to WT mice (Fig. 3*B*). However, at 2 wk postinfection, increased numbers of alveolar macrophages (CD11c^+^ CD11b^int^ Siglec F^hi^), neutrophils (CD11b^hi^ Gr-1^+^), and eosinophils (CD11c^−^ CD11b^lo/hi^ Siglec F^int^) were observed in the lungs of WT mice relative to CSE^−/−^ mice (Fig. 3*C*). At 6 wk postinfection, despite an overall reduction in myeloid cells, the neutrophil population from CSE^−/−^ mice was still significantly reduced in comparison to WT mice. However, the alveolar macrophage population in CSE^−/−^ mice was greater than that in WT mice (Fig. 3*D*). These data suggest that the presence of CSE promotes an excessive innate immune response, and are consistent with previous studies demonstrating that increased neutrophils exacerbate TB disease (27), and that H_2_S triggers neutrophil infiltration during septic shock (28).

Second, although no significant differences were observed in the CD4^+^ T cells, increased numbers of CD8^+^ T cells were observed in the uninfected CSE^−/−^ mice than in the WT mice (Fig. 3 *F* and *I*). At 2 and 6 wk postinfection, CD4^+^ and CD8^+^ T cell populations were significantly higher in the lungs of CSE^−/−^ mice than in WT mice (Fig. 3 *G*, *H*, *J*, and *K*). Notably, increased effector memory (CD62^lo^ CD44^hi^) and naïve T cells (CD62^hi^ CD44^lo^) were found in both CD4^+^ and CD8^+^ T cells in the lungs of the CSE^−/−^ mice at 2 and 6 wk postinfection (Fig. 3 *G*, *H*, *J*, and *K*). These findings suggest that CSE^−/−^ mice mount a stronger adaptive immune response to *Mtb* infection than WT mice.

Third, we examined Treg cells (CD25^+^FoxP3^−^ and CD25^+^FoxP3^+^) and the IFN-γ–producing T cell (Th1) populations and found that the number of CD4^+^CD25^+^FoxP3^−^ T cells were greater in the uninfected WT mice than in the CSE^−/−^ mice, with no differences observed in the CD8^+^CD25^+^FoxP3^−^ T cells (Fig. 3 *L* and *O*). For both CD4^+^ and CD8^+^ T cells, levels of IFN-γ were greater in the uninfected WT mice (Fig. 3 *L* and *O*). However, after 2 and 4 wk of infection, CSE^−/−^ mice showed greater numbers of CD25^+^FoxP3^−^ cells than WT mice in both CD4^+^ and CD8^+^ T cells (Fig. 3 *M*, *N*, *P*, and *Q*). Furthermore, at 2 wk postinfection, the number of CD4^+^CD25^+^FoxP3^+^ Treg cells was significantly lower in the lungs of CSE^−/−^ mice (Fig. 3*M*), which was reversed at 4 wk postinfection (Fig. 3*N*), suggesting control of the proinflammatory immune response in the CSE^−/−^ mice after 4 wk of infection. However, there were no significant differences in the numbers of CD8^+^CD25^+^ FoxP3^+^ Treg cells between the lungs of WT and CSE^−/−^ mice at 2 and 4 wk postinfection (Fig. 3 *P* and *Q*). Additionally, we found that the number of IFN-γ^+^ T cells was significantly higher in the lungs of CSE^−/−^ mice at 2 and 4 wk postinfection (Fig. 3 *M*, *N*, *P*, and *Q*). Increased numbers of IFN-γ–producing T cells in CSE^−/−^ mice are reflected by the increased number of effector memory T cells (Fig. 3 *G*, *H*, *J*, and *K*), decreased levels of neutrophils (27) (Fig. 3 *C* and *D*), and the subsequent control of *Mtb* growth in vivo (Fig. 2 *A*–*H*).

Fourth, we examined cytokine levels in the mouse serum and found that the levels of proinflammatory cytokines, IL-1β, IL-6, TNF-α, IL-12, IFN-γ, and IL-17, which have been previously described to control TB (29), were significantly increased in the serum of CSE^−/−^ mice after 2 or 3 wk of infection (Fig. 3*R*). Lower levels of the antiinflammatory cytokines, IL-10 and IL-13, after 2 wk of infection, followed by a significant increase in IL-10 levels after 3 wk of infection in CSE^−/−^ mice reflects controlling mechanisms of the proinflammatory response in CSE^−/−^ mice.

Altogether, our data indicate that CSE promotes an excessive innate immune response and reduces the adaptive immune response to *Mtb* infection in the lung. We show that reduced numbers of effector memory T cells in *Mtb* infection decrease the number of IFN-γ–producing T cells, which increases the levels of neutrophils that exacerbate disease. Furthermore, CSE decreases the production of circulating proinflammatory cytokines and cytokines necessary for the control of TB infection. Hence, our data point to CSE as a modulator of the innate and adaptive immunity and a potential pharmacological target that may lead to immune restoration and control of *Mtb* infection.

### Excessive Levels of CSE-Generated H_2_S Augments *Mtb* Growth in Macrophages by Impeding IL-1β, IL-6, and IL-12 Production.

**Fig. 4.**
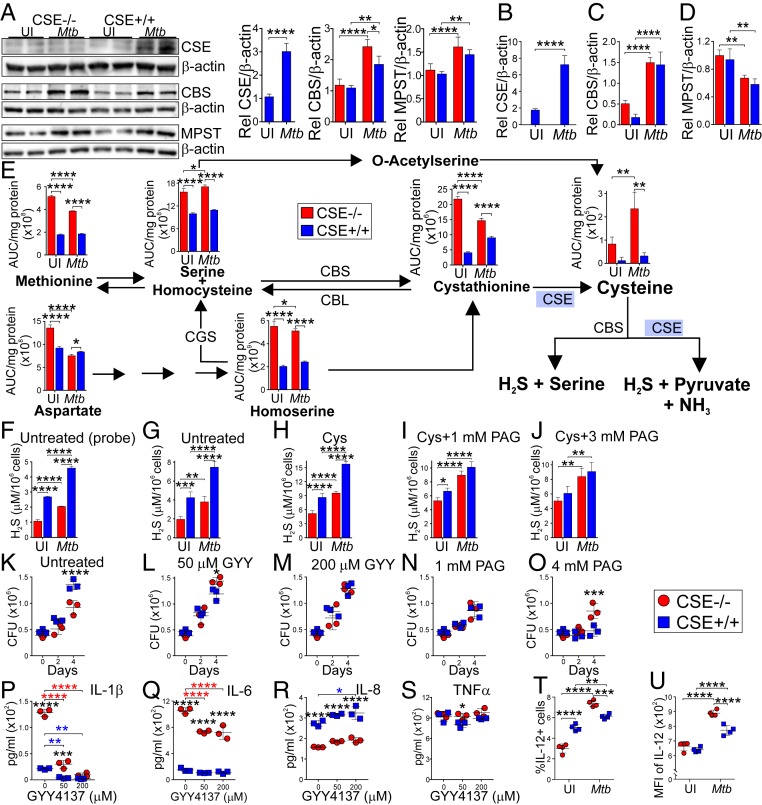
Endogenous H_2_S produced by CSE supports *Mtb* growth in macrophages. (*A*) Western blot showing expression of CSE, CBS, and MPST in uninfected and *Mtb*-infected peritoneal macrophages (PMs) at 24 h postinfection. Densitometric quantitation of the Western blot bands relative to the β-actin band intensities are shown in the bar graphs (*Right*). Expression in the uninfected WT peritoneal macrophages has been normalized to 1. Transcription of the (*B*) *CSE*, (*C*) *CBS*, and (*D*) *MPST* genes in uninfected and *Mtb*-infected PMs relative (Rel) to transcription of the β-actin gene at 24 h postinfection. (*E*) LC-MS/MS quantitation of amino acids involved in the endogenous H_2_S pathway in uninfected and *Mtb*-infected PMs at 24 h postinfection. (*F*–*J*) Measurement of H_2_S in the supernatants of uninfected and *Mtb*-infected PMs at 24 h postinfection using a probe-based H_2_S microsensor (*F*), and the conventional methylene blue method (*G*) of untreated and 4-h treatments with (*H*) 2 mM Cys, (*I*) 2 mM Cys and 1 mM PAG (an irreversible and specific inhibitor of CSE), (*J*) 2 mM Cys and 3 mM PAG. (*K*) Bacterial burden of *Mtb*-infected PMs at day 0, day 2, and day 4 postinfection, after treatment with (*L*) 50 µM GYY4137 (a slow releaser of H_2_S), (*M*) 200 µM GYY4137, (*N*) 1 mM PAG, and (*O*) 4 mM PAG. Error bars represent SD of the mean of four replicates. (*P*–*S*) Cytokine levels of (*P*) IL-1β, (*Q*) IL-6, (*R*) IL-8, and (*S*) TNF-α in the supernatants of *Mtb*-infected PMs at 24 h postinfection. Blue asterisks indicate significance between WT PMs; red asterisks indicate significance between CSE^−/−^ PMs; black asterisks indicate significance between WT and CSE^−/−^ PMs. (*T*–*U*) Intracellular measurements of IL-12 production of uninfected and *Mtb*-infected PMs at 24 h postinfection. Two-way ANOVA was used to determine statistical significance of all of the data. Error bars represent SD of the mean of three to four biological replicates. Data are representative of two independent experiments; ****P < 0.0001; ***P < 0.001; **P < 0.01; *P < 0.05.

Next, we asked whether CSE-derived H_2_S regulates *Mtb* growth in macrophages and modulates sulfur metabolites to regulate cytokine production. First, we validated that the intraperitoneal macrophages generated from the CSE^−/−^ mice did not express the CSE protein (Fig. 4*A*) nor transcribe the *CSE* gene (Fig. 4*B*). Furthermore, there was no compensation in the transcription (Fig. 4 *C* and *D*) or the expression (Fig. 4*A*) of CBS and MPST in both of the uninfected WT and CSE^−/−^ macrophages. Interestingly, following infection, the production of CSE increased in the WT, whereas the production and transcription of CBS increased in both the WT and CSE^−/−^ macrophages (Fig. 4 *A* and *C*), with significantly greater CBS expression in the infected CSE^−/−^ macrophages (Fig. 4*A*). MPST protein levels were also increased in both WT and CSE^−/−^ macrophages after infection (Fig. 4*A*); however, the transcription of *MPST* decreased after infection (Fig. 4*D*).

Second, we investigated the levels of the sulfur metabolites in the macrophages. H_2_S is produced endogenously from Cys, which is produced through various pathways involving methionine (Met), homocysteine (Hcy), serine (Ser), homoserine (Hse), cystathionine (Cth), and Cys (Fig. 4*E*). Infection of CSE^−/−^ mouse macrophages with *Mtb* followed by LC-MS/MS analysis of the intermediates in the H_2_S biosynthesis pathway demonstrate that all metabolites were increased in uninfected and infected CSE^−/−^ macrophages compared to the WT (Fig. 4*E*), except for Asp. Unlike the WT macrophages, *Mtb* infection increased the Cys and Ser levels and decreased other metabolites in CSE^−/−^ macrophages.

Since CSE can exert pleiotropic effects, we first confirmed that H_2_S was the effector molecule using a highly sensitive H_2_S microprobe technology to specifically quantify H_2_S. We found that WT cells produce more H_2_S in uninfected and infected cells than CSE^−/−^ cells (Fig. 4*F*), which was confirmed using the conventional methylene blue technique measuring the sulfide pool (H_2_S + HS^−^ + S^2−^) (Fig. 4* G–J*). Furthermore, addition of the CSE substrate Cys increased the overall levels of H_2_S production (Fig. 4*H*), whereas progressive inhibition of CSE using the inhibitor DL-propargylglycine (PAG) resulted in reduced H_2_S levels produced by WT cells (Fig. 4 *H–J*). These results support the conclusion that *Mtb* infection of WT macrophages increases H_2_S levels to detrimental levels for the host.

We next examined the contribution of H_2_S toward *Mtb* survival in macrophages. Our time-dependent in vitro colony forming unit (CFU) results showed that infected CSE^−/−^ macrophages had reduced CFUs after 4 d of infection compared to WT macrophages (Fig. 4*K*), which is consistent with our in vivo data (Fig. 2 *B*–*D*). To demonstrate that H_2_S is the effector molecule, we chemically complemented infected CSE^−/−^ macrophages with different concentrations of exogenously added H_2_S using GYY4137, which releases low amounts of H_2_S over a sustained period to mimic physiological production (24). Addition of GYY4137 to CSE^−/−^ macrophages increased CFUs, resembling that of infected WT macrophages (Fig. 4 *L* and *M*). Control experiments using spent (decomposed) GYY4137 lacking the H_2_S donor group confirmed that the chemical backbone did not have any effects on mycobacterial growth in the macrophages (*SI Appendix*, Fig. S11). Also, addition of the CSE inhibitor PAG to infected WT macrophages significantly reduced *Mtb* CFUs to levels comparable (Fig. 4*N*) or even lower (Fig. 4*O*) than that of CSE^−/−^macrophages. The latter can be explained since PAG, like many other inhibitors, has some degree of nonspecificity (24). Overall, our chemical complementation and inhibition data provide evidence that H_2_S is the effector molecule regulating *Mtb* survival in macrophages.

Next, we examined the role of CSE in macrophage cytokine production. Previously, IL-1β was shown to play a major role in host resistance to *Mtb* infection (30), and similar roles were described for IL-6, IL-8, and TNF-α (29). Increased levels of IL-1β and IL-6, and reduced levels of IL-8 were found in the supernatants of *Mtb*-infected CSE^−/−^ macrophages compared to that of the WT macrophages (Fig. 4 *P*–*R*), whereas no significant differences were observed in TNF-α levels (Fig. 4*S*). Notably, exogenous addition of H_2_S using GYY4137 significantly reduced IL-1β and IL-6, but not IL-8 levels of the infected CSE^−/−^ macrophages toward infected WT levels (Fig. 4 *P*–*R*). Cytokines produced from uninfected mouse macrophages were below the limit of detection. Since we have shown that CSE modulates innate and adaptive immunity (Fig. 3), we considered IL-12 in macrophages in vitro, which connects the innate and adaptive host responses to *Mtb* (29). Notably, the percentage and mean fluorescence intensity (MFI) of infected CSE^−/−^ macrophages producing IL-12 was greater than that of WT macrophages (Fig. 4 *T* and *U*). This is consistent with the increased numbers of IFN-γ–producing T cells observed in vivo in the infected CSE^−/−^ mice (Fig. 3 *L*–*Q*). These findings support the conclusion that H_2_S regulates IL-12 that controls IFN-γ (29), which down-regulates neutrophil infiltration (27) (Fig. 3 *C* and *D*). In addition, the decreased levels of IL-8 observed in the CSE^−/−^ macrophages (Fig. 4*R*) also correlates with the lower numbers of neutrophils in the infected CSE^−/−^ mice (Fig. 3 *C* and *D*).

In sum, our metabolomic data demonstrate that CSE is essential for maintaining homeostatic levels of key sulfur substrates in the CSE pathway responsible for generating H_2_S, the levels of which are perturbed during *Mtb* infection. Our chemical complementation and CSE inhibition studies suggest that CSE-generated H_2_S is the effector molecule responsible for the lack of control of intracellular bacillary growth in macrophages. This occurs via the down-regulation of key cytokines, such as IL-1β, IL-6, and IL-12 in the host response to *Mtb*.

### Excessive H_2_S Production Stimulated by *Mtb* Infection Inhibits Glycolysis and Cellular Respiration.

Having established that human TB lungs show increased CSE and MPST levels (Fig. 1 and *SI Appendix*, Figs. S1–S5), and that infected WT macrophages produce supraphysiological levels of H_2_S (Fig. 4 *F*–*J*), it is reasonable to hypothesize that excessive levels of H_2_S impairs bioenergetic homeostasis of the infected cell to exacerbate disease. This would dampen immunity as is evident from our IL-1β, IL-6, and IL-12 mouse findings (Fig. 4 *P*, *Q*, *T*, and *U*). We tested this hypothesis by infecting macrophages from CSE^−/−^ and WT mice with *Mtb*, performing transcriptomic analysis on the RNA isolated from the macrophages, and measuring the rates of glycolysis and mitochondrial respiration using an XF96 Extracellular Flux Analyzer (Agilent), previously adapted by us (21, 22, 31).

**Fig. 5.**
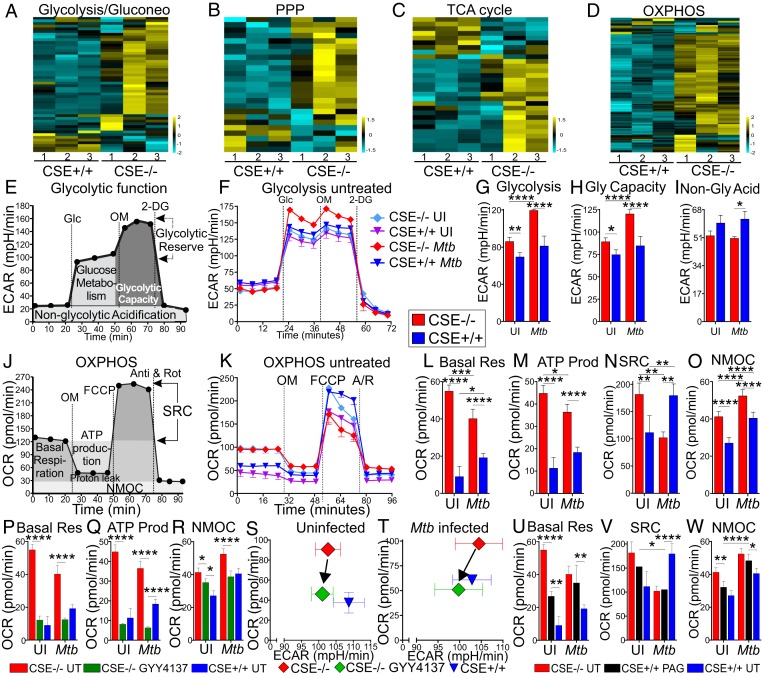
CSE suppresses cellular respiration in *Mtb*-infected macrophages. Heatmaps of RNA-seq data of *Mtb* infected PMs 24 h postinfection, showing differential expression of genes involved in (*A*) glycolysis and gluconeogenesis, (*B*) PPP, (*C*) TCA cycle, (*D*) OXPHOS pathway, *n* = 3. (*E*) Schematic representation of glycolysis stress test XF assay to measure ECAR and glycolytic function. Glc, glucose; OM, oligomycin; 2-DG, 2-deoxyglucose. (*F*) ECAR profiles of uninfected and *Mtb*-infected peritoneal macrophages 24 h postinfection and their glycolytic parameters: (*G*) glycolytic ECAR, (*H*) glycolytic capacity, and (*I*) nonglycolytic acidification. (*J*) Schematic representation of the Cell Mito Stress Test XF assay to measure OCR and mitochondrial respiration (OXPHOS). FCCP, carbonyl cyanide-p-trifluoromethoxyphenylhydrazone; A/R, antimycin A and rotenone. (*K*) OCR profiles of uninfected and *Mtb*-infected PMs 24 h postinfection and their respiratory parameters: (*L*) basal respiration (Basal Res), (*M*) ATP production OCR (ATP Prod), (*N*) SRC (spare respiratory capacity), and (*O*) NMOC (nonmitochondrial respiration). (*P*–*R*) Basal Res, ATP Prod, and NMOC of CSE^−/−^ PMs treated with 50 μM GYY4137 for 24 h compared to untreated CSE^−/−^ and WT (CSE^+/+^) PMs. (*S* and *T*) Plots of OCR versus ECAR of uninfected (*S*) and *Mtb*-infected (*T*) PM after treatment with 50 μM GYY4137 for 24 h. (*U*–*W*) Basal Res (*U*), SRC (*V*), and NMOC (*W*) of CSE^+/+^ PMs treated with 1 mM PAG for 24 h compared to untreated CSE^−/−^ and WT PMs. Error bars represent SD from the mean of three to five replicates. Two-way ANOVA was used to determine statistical significance. All data are representative of two independent experiments; ****P < 0.0001; ***P < 0.001; **P < 0.01; *P < 0.05.

First, transcriptomic analysis revealed an increase in the expression of the genes related to central carbon metabolism, in particular, glycolysis (Fig. 5*A*), the pentose phosphate pathway (Fig. 5*B*), the TCA cycle (Fig. 5*C*), and OXPHOS (Fig. 5*D* and *SI Appendix*, Fig. S12) of the infected CSE^−/−^ macrophages relative to that of the WT cells. These significant differences were not observed between the uninfected CSE^−/−^ and WT macrophages (National Center for Biotechnology Information Gene Expression Omnibus, GSE143619).

Second, glycolysis was assessed with the glycolysis stress test and monitoring of extracellular acidification rate (ECAR). ECAR was measured before and after sequential injections of glucose, oligomycin, and 2-deoxyglucose, and used to calculate nonglycolytic acidification, glycolysis, and glycolytic capacity (Fig. 5*E*). The rate of glycolytic acidification, ECAR, following the addition of glucose (Glc) (Fig. 5*F*), and the glycolytic capacity determined from ECAR after addition of oligomycin to inhibit mitochondrial ATP synthase, were significantly higher in CSE^−/−^ mouse macrophages than in WT macrophages (Fig. 5 *G* and *H*). Nonglycolytic acidification (Fig. 5*I*) was greater in the WT macrophages after *Mtb* infection, suggesting that more NADH and carbonic acid are produced in *Mtb*-infected WT macrophages by the TCA cycle. Since increased glycolysis is characteristic of proinflammatory macrophages (32–34), these data underscore the conclusion that *Mtb* infection of WT mice triggers excessive H_2_S production that decelerates glycolysis, leading to increased organ burden (Fig. 2 *B*–*D*) and reduced mouse survival (Fig. 2*A*).

Third, we analyzed mitochondrial respiration by measuring the oxygen consumption rate (OCR) in macrophages from CSE^−/−^ and WT mice using the Cell Mito Stress Test. OCR measured before and after sequential injections of oligomycin, carbonyl cyanide-4-(trifluoromethoxy)phenylhydrazone and antimycin/rotenone was used to calculate the basal respiration, ATP production OCR, spare respiratory capacity (SRC), and nonmitochondrial respiration (NMOC) (Fig. 5*J*). The data demonstrate significantly increased basal respiration, ATP production OCR, and NMOC in uninfected and infected CSE^−/−^ macrophages compared to WT macrophages (Fig. 5 *K*–*M* and *O*). This suggests that endogenous levels of H_2_S produced by uninfected and infected macrophages suppresses respiration. However, after *Mtb* infection, the SRC was lower in the CSE^−/−^ macrophages than in WT macrophages (Fig. 5*N*), indicating that these macrophages might be utilizing their SRC for basal respiration and ATP production during infection.

To implicate CSE-generated H_2_S as the effector molecule, infected macrophages were treated with GYY4137 or PAG. Treatment of the CSE^−/−^ macrophages with GYY4137 reduced their basal respiration (Fig. 5*P*), ATP production OCR (Fig. 5*Q*), and NMOC (Fig. 5*R*) closer to that of the WT macrophages in both uninfected and infected cells. The reduction in ATP production OCR in the GYY4137-treated CSE^−/−^ macrophages below that of WT is likely due to imprecise levels of H_2_S generated by GYY4137. Plots of OCR against ECAR prior to the addition of any inhibitors revealed how the addition of GYY4137 redirected the metabolic phenotype of the CSE^−/−^ macrophages to that of the WT macrophages (Fig. 5*S*), which was also evident after *Mtb* infection (Fig. 5*T*). Treatment of the uninfected WT macrophages with the CSE inhibitor PAG increased their basal respiration (Fig. 5*U*), but not the SRC (Fig. 5*V*) or the NMOC (Fig. 5*W*), closer to that of the CSE^−/−^ macrophages. However, chemical complementation was observed in the *Mtb*-infected macrophages, with PAG treatment of the WT macrophages increasing their basal respiration (Fig. 5*U*), reducing their SRC (Fig. 5*V*), and increasing their NMOC (Fig. 5*W*) to levels almost identical to that of the infected CSE^−/−^ macrophages. These data demonstrate that the absence or enzymatic inhibition of CSE increases glycolysis and mitochondrial respiration during infection, whereas the presence of CSE or addition of exogeneous H_2_S depresses mitochondrial respiration. These findings point to H_2_S as a key regulator of central energy metabolism during *Mtb* infection.

### CSE Suppresses Glycolysis and the Pentose Phosphate Pathway in *Mtb*-Infected Macrophages.

**Fig. 6.**
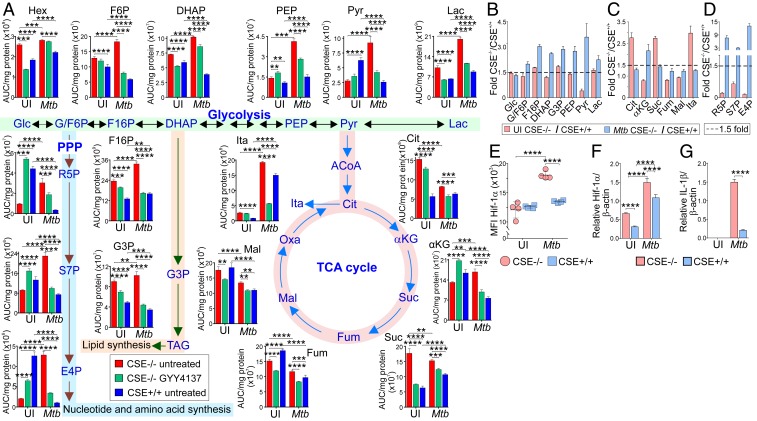
CSE controls glycolysis and the pentose phosphate pathway. (*A*) Normalized levels of metabolites of glycolysis, the PPP, and the TCA cycle in uninfected and *Mtb*-infected PM 24 h postinfection that are treated or not with 50 µM GYY4137. Red bars, CSE^−/−^ untreated; green bars, CSE^−/−^ treated with 50 µM GYY4137; and blue bars, WT untreated. (*B*–*D*) Fold-change in metabolite levels of (*B*) glycolysis, (*C*) the TCA cycle, and (*D*) the PPP of uninfected (red) and *Mtb*-infected (blue) CSE^−/−^ versus WT PM. (*E*) Levels of intracellular Hif-1α, and RNA transcription of (*F*) Hif-1α and (*G*) IL-1β relative to the β-actin gene in uninfected and *Mtb*-infected PM 24 h postinfection. Error bars represent SD from the mean of four biological replicates. Two-way ANOVA was used to determine statistical significance. Data are representative of two independent experiments; ****P < 0.0001; ***P < 0.001; **P < 0.01.

To test the hypothesis that CSE regulates central carbon metabolism during *Mtb* infection, we infected WT and CSE^−/−^ macrophages with *Mtb* in the presence and absence of GYY4137 and used LC-MS/MS to analyze the metabolites. Overall, our metabolomic data support the conclusion that CSE-generated H_2_S during *Mtb* infection suppresses central carbon metabolism, which is supported by several lines of evidence. First, whereas most glycolytic metabolites were moderately increased in uninfected CSE^−/−^ macrophages, we found significantly enhanced levels of the glycolytic metabolites in infected CSE^−/−^ macrophages relative to WT macrophages (Fig. 6 *A* and *B*). This is further supported by the substantial increase in fructose-1,6-bisphosphate (F1,6P) in CSE^−/−^ vs. WT macrophages, which is the product of the rate-limiting enzyme for glycolysis, ATP-dependent phosphofructokinase. Furthermore, we observed an almost twofold increase in the levels of glycerol 3-phosphate (G3P) in CSE^−/−^ macrophages, which is essential for lipid synthesis (Fig. 6*B*). Second, of notable interest is that exogenous supplementation of H_2_S using GYY4137 to CSE^−/−^ macrophages is capable of partially or fully decreasing most glycolytic metabolite levels to that of WT macrophages (Fig. 6*A*).

The levels of the TCA metabolites (Fig. 6 *A* and *C*) in uninfected CSE^−/−^ macrophages are representative of a broken TCA cycle, which appears to be restored upon *Mtb* infection. Interestingly, itaconic acid (ITA) levels were approximately threefold higher in uninfected CSE^−/−^ macrophages compared to WT macrophages. *Mtb* infection increased overall ITA levels compared to uninfected cells, but the relative ratio between infected CSE^−/−^ and WT macrophages was reduced to ∼1.3-fold (Fig. 6*C*), suggesting that other activation signals are involved in the modulation of ITA upon infection. ITA has been shown to inhibit succinate dehydrogenase, thereby increasing levels of succinate that promote the production of IL-1β via HIF-1α (35), which is essential for control of *Mtb* growth (30). Notably, the metabolic phenotype observed in the CSE^−/−^ macrophages is characteristic of the metabolic rewiring that occurs in proinflammatory macrophages (33), suggesting that the uninfected CSE^−/−^ macrophages are polarized toward a proinflammatory phenotype prior to infection. The increased level of succinate in infected CSE^−/−^ macrophages further supports the observed increased IL-1β secretion (Fig. 4*P*) and transcription (Fig. 6*G*) of infected CSE^−/−^ mouse macrophages.

As is evident from the elevated levels of ribose-5-phosphate (∼8-fold), sedoheptulose-7-phosphate (∼4-fold), and erythrose 4-phosphate (∼12-fold) in infected CSE^−/−^ macrophages (Fig. 6 *A* and *D*) compared to WT macrophages, the H_2_S produced by the WT cells suppresses the pentose phosphate pathway (PPP) after infection. In contrast, PPP metabolites were significantly reduced in uninfected CSE^−/−^ macrophages compared to WT macrophages (Fig. 6 *A* and *D*). Again, chemical complementation of CSE^−/−^ macrophages with H_2_S shifted the levels of PPP metabolites toward that of WT macrophages. Finally, since glycolysis and IL-1β secretion are regulated by HIF-1α, we examined whether HIF-1α levels increased with glycolytic flux. Indeed, infected CSE^−/−^ cells shows increased HIF-1α production and expression (Fig. 6 *E* and *F*).

Overall, increased levels of glycolytic and PPP metabolites were observed in *Mtb* infected CSE^−/−^ macrophages compared to WT macrophages (Fig. 6 *B* and *D*). The higher rates of glycolysis serve as a mechanism to rapidly produce ATP to sustain the high secretory and phagocytic functions of the macrophage, and to feed intermediates into the PPP. Importantly, the increased glycolytic flux in infected CSE^−/−^ cells is consistent with increased levels of succinate (Fig. 6 *A* and *C*), HIF-1α (Fig. 6 *E* and *F*), and IL-1β (Figs. 3*R*, 4*P*, and 6*G*). Enhanced PPP activity boosts production of NADPH for reactive oxygen intermediate (ROI) production to kill bacteria. NADPH also plays a role in lipogenesis, thus supporting the widely known role of generation of cellular organelles such as endoplasmic reticulum and Golgi bodies needed for cytokine biosynthesis.

Altogether, these data provide evidence that CSE-generated H_2_S suppresses glycolysis and the PPP during *Mtb* infection. Given that these pathways play a central role in cellular proliferation and immune activation, the capability of H_2_S to regulate these metabolic pathways is expected to have implications for understanding how *Mtb* causes disease and persists long term.

### CSE Depletes Mitochondrial Mass, Modulates ROI, and Depolarizes the Mitochondrial Membrane Potential in *Mtb* Infected Macrophages.

**Fig. 7.**
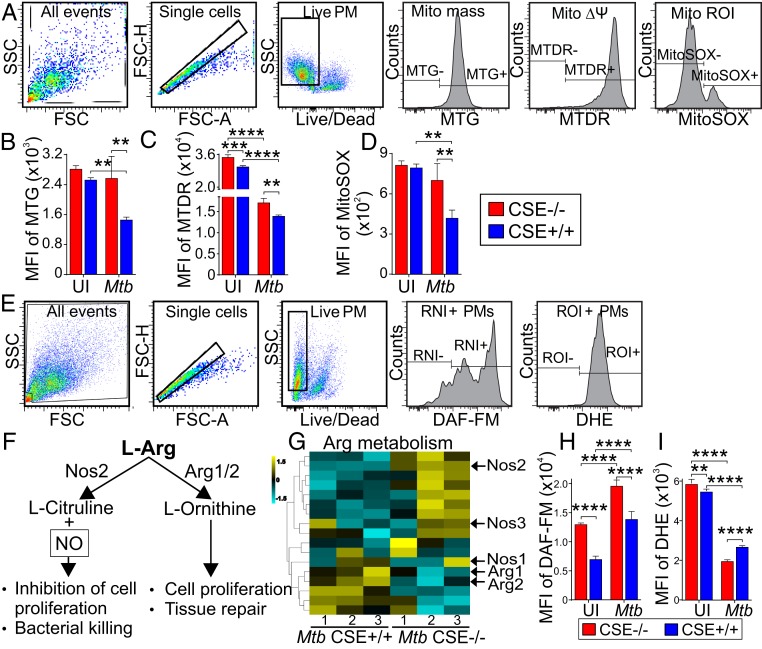
CSE regulates NO and ROI production after *Mtb* infection. (*A*) Gating strategies and MFI of (*B*) MitoTracker green (MTG), for measurement of mitochondrial mass, (*C*) MitoTracker Deep Red (MTDR) for measurement of MMP, and (*D*) MitoSOX red for measurement of mROI of uninfected and *Mtb*-infected PMs 24 h postinfection. (*E*) Gating strategies for measurement of cellular nitric oxide (NO, DAF-FM) and reactive oxygen intermediates (ROI, DHE). (*F*) Pathways of Arg metabolism, of which one generates NO for bacterial killing, and (*G*) the heatmap of RNA-seq data of enzymes involved in Arg metabolism. MFI of (*H*) DAF-FM for measurement of RNI (of which NO is the main constituent), and (*I*) DHE for measurement of ROI in uninfected and infected PMs 24 h postinfection. Error bars represent SD from the mean of four replicates. Two-way ANOVA was used to determine statistical significance. Data are representative of two independent experiments; ****P < 0.0001; ***P < 0.001; **P < 0.01.

Having shown that CSE-generated H_2_S plays a key role in mitochondrial respiration and energy metabolism during *Mtb* infection, we tested the hypothesis that CSE modulates mitochondrial mass, mitochondrial membrane potential (MMP), and the production of mitochondrial ROIs (mROI) (Fig. 7*A*). We infected macrophages from CSE^−/−^ and WT mice with *Mtb* and determined the mitochondrial mass of the macrophages using MitoTracker Green FM, which stains mitochondria independently of their membrane potential. Similar mitochondrial contents were found in the uninfected macrophages from both CSE^−/−^ and WT mice (Fig. 7*B*). After *Mtb* infection, although the mitochondrial mass remained unchanged in CSE^−/−^ macrophages, it significantly decreased in WT mouse macrophages (Fig. 7*B*). Next, we studied the MMP using MitoTracker Deep Red FM, which accumulates in mitochondria in a manner dependent on their membrane potential (Δψ). Uninfected CSE^−/−^ macrophages showed a significantly more polarized Δψ than WT macrophages that decreased after *Mtb* infection but was still more polarized than that of infected WT macrophages (Fig. 7*C*). We also examined mROI production using MitoSOX and observed similar levels of mROI in the uninfected WT and CSE^−/−^ macrophages (Fig. 7*D*). However, after *Mtb* infection, the levels of mROI did not change in the CSE^−/−^ macrophages but were significantly lower in WT macrophages (Fig. 7*D*).

Finally, we examined the role of CSE in cellular reactive nitrogen intermediates (RNI) and ROI production in *Mtb*-infected macrophages (Fig. 7*E*). iNOS (NOS2) catalyzes the production of NO from l-arginine as depicted in Fig. 7*F*. Arginine is alternatively used to produce l-ornithine in a reaction catalyzed by arginase. Transcriptomic analysis of genes involved in arginine metabolism indicated increased expression of *NOS1*, *NOS2*, *NOS3*, and reduced expression of *ARG1* and *ARG2* in the *Mtb*-infected CSE^−/−^ macrophages (Fig. 7*G*). Upon infection, RNI was significantly increased in both CSE^−/−^ and WT mouse macrophages; however, RNI was significantly higher in CSE^−/−^ macrophages than in WT cells (Fig. 7*H*). In contrast, cellular ROI was significantly reduced in both CSE^−/−^ and WT macrophages after *Mtb* infection, with significantly lower levels in the *Mtb* infected CSE^−/−^ macrophages (Fig. 7*I*).

Overall, we observed higher mitochondrial mass, Δψ, and mROI production, higher RNI production, but lower cellular ROI production in CSE^−/−^ macrophages than in WT macrophages. These data suggest that CSE-generated H_2_S plays an important role in regulating mitochondrial biogenesis, bioenergetics, mROI signaling, and RNI production to exacerbate *Mtb* disease. These findings, together with enhanced glycolysis (Figs. 5 *F*–*I* and Fig. 6 *A* and *B*), higher IFN-γ (Fig. 3 *L*–*R*), IL-1β (Figs. 3*R*, 4*P*, and 6*G*), and HIF-1α production (Fig. 6 *E* and *F*) support the observed *Mtb* growth control and enhanced bacterial killing in CSE^−/−^ mice compared to WT mice.

## Discussion

The major conclusion of the present study is that *Mtb* infection triggers supraphysiological levels of CSE-generated H_2_S that is associated with suppressed central carbon catabolism, in particular glycolysis and the PPP. Depressed glycolysis reduces the production of IL-1β and the levels of HIF-1α, which correlates with *Mtb* growth. This conclusion is supported by our animal studies demonstrating that reduced levels of H_2_S decrease organ burden and associated pathophysiology, and promote mouse survival by decreasing myeloid cell populations, increasing lymphoid cell populations, and increasing levels of circulating proinflammatory cytokines and cytokines that control TB. Consistent with these findings, we showed that H_2_S is associated with decreased mitochondrial biogenesis and ROI levels that may further support *Mtb* growth. Chemical complementation and CSE inhibitor experiments in macrophages identified H_2_S as the effector molecule. Finally, the clinical relevance of our findings was confirmed by examining the distribution of H_2_S-producing enzymes in human pulmonary TB tissues representing a spectrum of lesions. This represents a significant advancement over studies that rely solely on animal models that do not represent the full pathological spectrum of human TB. Overall, our data show excessive levels of H_2_S are associated with repressed central carbon metabolism, including reduced glycolysis and PPP that consequently down-regulates the HIF-1α levels and production of IL-1β during *Mtb* infection. Hence, our findings highlight the H_2_S-producing enzyme CSE as a potential therapeutic target to restrain TB disease. For example, D-Penicillamine (Cuprimine), which is commonly used to treat rheumatoid arthritis, targets CSE (36).

H_2_S exerts a wide variety of biological pleiotropic functions because of its biphasic character. At low local levels of H_2_S, multiple cytoprotective, antioxidant, and antiinflammatory functions can be exerted, whereas at higher local concentrations, this gas can become cytotoxic, cytostatic, and pro-oxidant (12–16, 23, 24, 37). Indeed, using an analytical microsensor for direct measurement of H_2_S, as well as an established technique for measuring total sulfide, we demonstrated that *Mtb* infection significantly increases H_2_S levels in WT macrophages relative to CSE^−/−^ macrophages. Our in vitro findings are consistent with the in vivo experiments where we discovered that CSE^−/−^ mice are more resistant to *Mtb* infection. This was evident by increased survival, and reduced organ burden and pathology compared to the WT mice. Overall, these in vivo findings strongly suggest that excessive H_2_S exacerbates TB disease.

How does excessive H_2_S exacerbate TB disease? Clinical studies using antiinflammatory drugs have demonstrated that *Mtb* infection triggers disproportionate inflammation in TB patients (38). Hence, there is substantial interest in pharmacological control of excessive inflammation. Our characterization of immune cell populations and serum cytokines in the WT and CSE^−/−^ mice supports a mechanism whereby excessive H_2_S promotes increased innate immunity, and down-regulates adaptive immunity, ultimately leading to increased TB disease. This modulation of immunity is also supported by decreased levels of cytokines responsible for controlling bacterial infections, such as IL-1β, IL-6, IL-9, IL-12, TNF-α, IL-17, and IFN-γ (29, 39), observed in the serum of the WT mice. The data point to H_2_S as a regulator of innate and adaptive immunity during *Mtb* infection and our findings agree with studies showing that CSE-deficient mice are resistant to sepsis and associated inflammatory responses (16).

In contrast to well-established, fundamental immune mechanisms for TB disease, new immunometabolic mechanisms that could lead to the pharmacological control of TB are urgently needed. Hence, establishing a role for H_2_S in the immunometabolism of TB may fill such a knowledge gap and may lead to new paradigms for intervention. The widely known role of H_2_S in modulating bioenergetics prompted us to investigate the effect of H_2_S on host bioenergetics during *Mtb* infection. Using a real-time, noninvasive approach that we have recently optimized for studying how *Mtb* reprograms host metabolism (21), as well as metabolomics, we found that excessive H_2_S significantly suppressed glycolysis. Notably, basal respiration, ATP production OCR, and SRC are suppressed in WT macrophages in comparison to CSE^−/−^ macrophages, which points toward distinct reduction of OXPHOS.

The stimulatory or inhibitory effects of H_2_S on mitochondrial respiration are complex and are ultimately dictated by the sulfide levels. At low levels, H_2_S acts as a substrate that binds and reduces cytochrome C oxidase (Cco) (37), whereas at higher levels, H_2_S inhibits respiration through Cco (40, 41). The two redox active metal sites, heme *a* and Cu_A_, and the binuclear center comprised of heme *a3* and Cu_B_ in Cco, play critical roles in the stimulatory or inhibitory effects of H_2_S on respiration. CO (2, 42) and NO (43), which are both implicated in TB dormancy, can also inhibit respiration, but do so under different conditions (44, 45). Notably, since Cco represents a critical metabolic checkpoint involved in life-and-death decisions early in T cell activation and differentiation (46), inhibition or stimulation of respiration by H_2_S has significant implications for cellular survival during *Mtb* infection. Furthermore, it has been shown that *Mtb* infection rewires metabolism to fuel fatty acid synthesis and so results in reduced OXPHOS in macrophages (21).

How is central metabolism linked to dysfunctional immunity? Several metabolites contribute directly to immunometabolism. For example, citrate is used for the biosynthesis of fatty acids (47) and influences the control of glycolysis via hexokinase 2, PFK1, and LDHA (48). Furthermore, succinate, which is increased in infected CSE^−/−^ macrophages promotes the production of the proinflammatory cytokine, IL-1β, and supports the stabilization of HIF-1α (35, 49, 50). The HIF pathway has been shown to function as a critical switch through which metabolic phenotypes can be regulated and HIF-1α has been shown to regulate multiple enzymes in the glycolytic pathway (32, 50). In agreement with this, we found the up-regulation of the PPP in infected CSE^−/−^ macrophages, which releases large quantities of NADPH used by NADPH oxidase to generate ROI for HIF-1α stabilization (32, 51). The increased glycolytic flux observed in infected CSE^−/−^ macrophages supports the increased levels of proinflammatory cytokines, IL-1β and IL-6, in addition to IL-12, which links innate and adaptive immunity, secreted by the macrophages and observed in the mouse serum (34, 52). It is well established that IFN-γ activation of macrophages is necessary to restrict *Mtb* growth. This is supported by our findings, which demonstrate that increased levels of IFN-γ produced in the lungs of infected CSE^−/−^ mice result in lower organ burdens. IFN-γ–mediated macrophage activation is important for HIF-1α–directed immunity against *Mtb* as a large number of IFN-γ–regulated genes in infected macrophages are under HIF-1α control (52). However, reduced levels of IL-8, which is involved in recruiting neutrophils, were secreted by the infected CSE^−/−^ macrophages in vitro. Furthermore, increased levels of circulating IL-10 in the CSE^−/−^ mouse serum were found after 3 wk of infection. These findings suggest a controlled inflammatory response that activates the adaptive immune response to clear the infection. In contrast, early production of excessive IL-10 and IL-13 antiinflammatory cytokines observed in the serum of WT mouse after 2 wk of infection promoted *Mtb* growth.

Finally, our mitochondrial data provide further evidence that in infected CSE^−/−^ macrophages, reduced H_2_S levels increased the levels of HIF-1α via increased ROI levels, consistent with published literature (53, 54). Overall, these findings demonstrate how excessive H_2_S rewires central metabolism and respiration during infection with an intracellular pathogen such as *Mtb*. A key finding is that excess H_2_S during *Mtb* infection reduces glycolysis. Glycolysis was previously shown to be important for the control of TB (34, 55) and recent stable isotope and real-time bioenergetic studies have shown that live *Mtb* decelerates glycolysis (21). This deceleration was likely a result of increased H_2_S production during *Mtb* infection. This argues that stimulating glycolysis will lead to the effective control of *Mtb* infection (34, 55). Hence, our findings suggest we have identified an effector molecule, H_2_S, which could be pharmacologically manipulated by D-Penicillamine to regulate glycolysis. Furthermore, it can be asked: How does H_2_S regulate metabolism? Naturally, the lipophilic and gaseous properties of H_2_S adds to the complexity of identifying the exact molecular targets of H_2_S. Nonetheless, recent studies have shown that H_2_S targets multiple enzymes in the glycolytic pathway through S-sulfhydration to modulate their activity (23), and regulates glucose uptake (56) and HIF-1α (53, 54). Hence, our findings that H_2_S regulates glycolysis and the PPP during *Mtb* infection are consistent with these published studies. Although it could be argued that H_2_S also regulates the TCA cycle, our data (Fig. 6 *A*–*D*) demonstrate that upon infection only α-ketoglutarate is differentially regulated compared to all of the glycolytic metabolites. Overall, our data suggest that glycolysis and the PPP are the predominant pathways influenced by H_2_S.

iNOS (5) and HO-1 (2, 5), which play essential roles in the control of TB, have been shown to be produced in the lungs of TB patients (2). In contrast, characterization of the lesional distribution of H_2_S-producing enzymes within the pathological spectrum pulmonary TB is unknown. Why is this important? First, as described recently for HO-1 (2), demonstrating H_2_S-producing enzymes within the microenvironment of the human TB lung provides clinical relevance, and contextualizes the spatial distribution of these enzymes. This is essential, as interpretation of positive immunohistochemical (IHC) signals depends on the microanatomic distribution and, hence, sample choice, which could be easily missed if a spectrum of lesions is not examined. Furthermore, the range of lesions, such as nonnecrotic and necrotic granulomatous lesions, of which the latter is hypoxic, is bound to affect the enzymatic activity of H_2_S-producing enzymes. For example, mammalian cells can utilize sulfide as a bioenergetic fuel, but also produce H_2_S during cellular stress (9, 37). Furthermore, H_2_S-producing enzymes do not require O_2_ as cofactor. Therefore, during hypoxia, which almost certainly exists in human TB lesions, the inhibitory effect of H_2_S on the electron transport chain is amplified (9) and will further promote disease. The strong CSE and MPST signals within the TB lungs raises the provocative idea that H_2_S could also function as an in vivo fuel source for *Mtb*. Not surprisingly, as was demonstrated in this study, overproduction of H_2_S is also associated with several other clinicopathological manifestations, including sepsis and associated inflammation (12–16). As was previously demonstrated in different inflammatory models, increased levels of H_2_S inhibit production of proinflammatory mediators, such as IL-1β, IL-6, TNF-α, NO, and mitochondrial ROI, but stimulate production of the antiinflammatory cytokine IL-10 in a dose-dependent manner (57–59), which supports our observations in *Mtb*-infected WT mice.

Linking the human TB microanatomic architecture to the spatial distribution of H_2_S-producing enzymes may help identify alternative therapeutic strategies and will provide a benchmark for validation of animal models of disease. Furthermore, given the widely known cytotoxicity of excessive H_2_S, the dysregulated accumulation of immune cells producing a highly diffusible gas at the site of infection illustrates how under one set of circumstances, homeostatic levels of H_2_S are beneficial, whereas in the case of human pulmonary TB, excessive levels of H_2_S are likely to be harmful.

One potential limitation of our study is that the CSE inhibitor PAG, has a degree of nonspecificity (24), which limits potency. However, using a microsensor that accurately measures H_2_S levels, we demonstrated that PAG reduces the levels of H_2_S produced by the WT macrophages. Furthermore, it is recognized that a whole-body CSE knockout mouse strain may trigger pleiotropic effects unrelated to H_2_S. However, this has been addressed to some extent, first, by chemical complementation using the widely used H_2_S donor, GYY4137, which mimics physiological release of H_2_S, and second by the CSE inhibitor, PAG. Finally, in our human pathology studies, a larger human cohort may produce a more divergent disease spectrum of H_2_S producing enzymes, which is the focus of ongoing studies.

In conclusion, our data demonstrate an unusual role for CSE-generated H_2_S in *Mtb* virulence and pathogenesis. The evidence shows that excessive H_2_S exacerbates *Mtb* disease by impairing central carbon catabolism and dysregulating the immune response. These responses include the regulation of glycolysis, the PPP, proinflammatory cytokines, and HIF-1α, as well as mROI-mediated stress, which overall dictate TB immunopathology (*SI Appendix*, Fig. S13). We have also demonstrated the clinical relevance of H_2_S-producing enzymes in human pulmonary TB tissue. Finally, the unusual role CSE-generated H_2_S plays in *Mtb* virulence and pathogenesis suggest that pharmacological inhibition of CSE (e.g., the antiarthritis drug D-Penicillamine) may limit lethal immunopathology during TB.

## Materials and Methods

All animal experiments were approved by the University of KwaZulu-Natal Animal Research Ethics Committee (Protocol reference no.: 125/14/Animal), the human lung pathology study was approved by the University of KwaZulu-Natal Biomedical Research Ethics Committee (Class approval study no. BCA 535/16), and patients undergoing lung resection for TB were approved by the University of KwaZulu-Natal Biomedical Research Ethics Committee (Study ID: BE 019/13). For in vitro and in vivo infection experiments, histopathology, cytokines, H_2_S measurements, metabolite analysis, bioenergetic experiments, and statistics, see *SI Appendix*.

### Data and Material Availability.

Raw RNA-seq sequence reads were uploaded to National Center for Biotechnology Information Gene Expression Omnibus (GSE143619). High-resolution immune staining figures are available from the corresponding author upon request. Please contact the corresponding author for more information.

## Supplementary Material

Supplementary File
