# Supplementary material for: Hydrogen sulfide dysregulates the immune response by suppressing central carbon metabolism to promote tuberculosis
Source: Proc Natl Acad Sci U S A. 2020 Mar 5;117(12):6663–74. doi: 10.1073/pnas.1919211117 (PMC7104411; doi:10.1073/pnas.1919211117)
Supplement: Supplementary File [file pnas.1919211117.sapp.pdf]

PNAS
www.pnas.org

Supplementary Information for:

Hydrogen sulfide dysregulates the immune response by suppressing central carbon metabolism to promote tuberculosis

Md. Aejazur Rahman[1], Bridgette M. Cumming[1], Kelvin W. Addicott[1], Hayden T. Pacl[3], Shannon L. Russell[1], Kievershen Nargan[1], Threnesan Naidoo[2], Pratistadevi K. Ramdial[2], John H. Adamson[1], Rui Wang[6], Adrie J. C. Steyn[1,3*]

[1]Africa Health Research Institute, Durban, KwaZulu Natal, South Africa,

[2]Department of Anatomical Pathology, National Health Laboratory Service, University of KwaZulu-Natal, Inkosi Albert Luthuli Central Hospital, Durban, South Africa,

[3]Department of Microbiology, University of Alabama at Birmingham, Birmingham, Alabama, United States of America,

[4]Centers for AIDS Research and Free Radical Biology, University of Alabama at Birmingham, Birmingham, Alabama, United States of America,

[5]School of Laboratory Medicine and Medical Sciences, Nelson R. Mandela School of Medicine, University of KwaZulu-Natal, Durban, South Africa

[6]Department of Biology, York University, Toronto, Canada

Corresponding author:
Adrie JC Steyn, PhD
asteyn@uab.edu

This PDF file includes:

Supplementary text
Figures S1 to S13
Table S1
SI References

www.pnas.org/cgi/doi/10.1073/pnas.1919211117

SI Appendix: Materials and Methods:

Ethics statement. All animal experiments were approved by the University of KwaZulu-Natal Animal Research Ethics Committee (Protocol reference number: 125/14/Animal). CSE$^{-/-}$ and WT mice were bred and maintained in our animal facilities and *Mtb* infected mice were maintained at our biosafety level-3 at the Africa Health Research Institute (AHRI), K-RITH Tower, Nelson R. Mandela School of Medicine, UKZN, Durban, South Africa in accordance with the guidelines set forth by the South African National Standard (SANS 10386:2008). Mice were maintained on standard rodent chow and had access to food and water. The human lung pathology study was approved by the University of KwaZulu-Natal Biomedical Research Ethics Committee (BREC, Class approval study number BCA 535/16). Patients undergoing lung resection for TB, their study protocol, associated informed consent documents, and data collection tools were approved by the UKZN BREC (Study ID: BE 019/13). Written informed consent was obtained from patients recruited from King DinuZulu Hospital Complex, a tertiary center for TB patients in Durban, South Africa.

Human lung histology. Human lung tissues were cut into 2 mm thick sections, mounted on charged slides and heated at 56°C for 15 min. Mounted sections were dewaxed in xylene followed by rinsing in 100% ethanol and 1 change of SVR (95% ethanol). Slides were then washed under running water for 2 min followed by antigen retrieval via Heat Induced Epitope Retrieval (HIER) in Tris-sodium chloride (pH 6.0) for 30 min. Slides were then cooled for 15 min and rinsed under running water for 2 min. Endogenous peroxidase activity was blocked by incubating the slides in 3% hydrogen peroxide for 10 min at room temperature (RT). Slides were then washed in PBST and blocked with protein block (Novolink) for 5 min at RT. Sections were incubated with primary antibodies for CSE (NBP1-31759, Novus Biologicals, 1:500), CBS (H00000875-D01P, Novus Biologicals,1:1000), MPST (NBP1-82617, Novus Biologicals, 1:100) at 4°C overnight followed by washing and incubation with HRP conjugated goat anti-rabbit IgG HRP (ab6721, ABCAM) for 30 min at RT. Slides were then washed and stained with DAB for 5 min, washed under running water and counterstained with hematoxylin for 2

min. Slides were rinsed under running water, blued in 3% ammoniated water for 30 s, washed under water, dehydrated and mounted in Distyrene Plasticiser Xylene (DPX). For isotype control, a similar protocol was followed and rabbit IgG (ab37415, Abcam) was used (at the same concentration/dilution as the primary antibodies) in place of the primary antibodies (isotype control).

***Mtb* and Mice.** *M. tuberculosis* strain H37Rv were passaged in mice, frozen in Middlebrook 7H9 broth (Gibco) containing 20% glycerol and stored at −80°C. For each experiment, *Mtb* frozen stock was cultured in Middlebrook 7H9 broth (Gibco) supplemented with 10% (v/v) oleic acid-albumin-dextrose-catalase (OADC, Difco), 0.5% (v/v) glycerol and 0.01% (v/v) Tyloxapol (Sigma) at 37°C with shaking to an OD_{600} of 1.0. To infect mice, the *Mtb* culture at an OD_{600} of 1.0 was diluted 1:100 in 7H9 media with 10% OADC prior to aerosol challenge. All work with live *Mtb* culture, infected cells and mice were performed in a biosafety level-3 laboratory. $CSE^{-/-}$ and wild-type (C57BL/6J X 129SvEv) mice (1) were bred and maintained in the AHRI animal facility. All mice used in this study were age matched from 6 to 8 weeks. Genotypes of $CSE^{-/-}$ and WT ($CSE^{+/+}$) mice were confirmed by performing standard PCR on genomic DNA isolated (QIAamp DNA Blood Mini Kit, Qiagen) from blood drawn from the saphenous vein. PCR was performed using a three-primer set in two reactions, as described previously (1). The N1 primer (Table S1) is specific for the $CSE^{-/-}$ allele and the F1 primer (Table S1) is for WT ($CSE^{+/+}$) allele. The reverse primer, R1 (Table S1), is common for both the $CSE^{-/-}$ and WT alleles.

Mice infection, survival and CFU. Mice were infected with *Mtb* H37Rv (15 mL of 1 x 10^6 CFU) by the aerosol route using a Glas-Col inhalation exposure system. Bacilli numbers that reached the lungs of the mouse were determined by plating homogenized lungs on 7H11 Middlebrook plates 24 hours post infection and found to be approximately 200 CFU per lung in both the WT and $CSE^{-/-}$ mice. For all time-course infection studies (For studies over the course of infection), mice were sacrificed at 2, 4 and 6 weeks post-infection and the lungs,

spleen and liver were aseptically harvested from the mice and homogenised in 5 mL DPBS containing 0.05% Tween 80 using a gentleMACS™ Dissociator (Miltenyi Biotec). Viable *Mtb* were determined as CFU by plating serial dilutions of organ homogenates on Middlebrook 7H11 agar plates supplemented with 10% OADC, 0.5% glycerol, and PACT (Mycobacteria Selectatab (Kirchner): Polymyxin B 25 mg/L, Amphotericin B 10 mg/L, Carbenicillin 50 mg/L, and Trimethoprim 20 mg/L). Plates were incubated at 37°C with 5% CO_2 for 3-4 weeks to determine CFU counts. The number of CFU are presented as a dot-plot showing the number of CFU per organ per mouse from five mice for each group per time point. For survival studies, 14 *Mtb* infected mice were used per group and time-to-death was observed and presented as a Kaplan-Meier survival curve.

Mouse histopathology. Histological sections of *Mtb* infected or uninfected lungs were stained with either hematoxylin-eosin (H&E) or Ziehl-Neelsen (ZN or acid-fast) stains for evaluation of granulomatous inflammation or detection of *Mtb* bacillus, respectively. Briefly, mouse lung was harvested and fixed in 10% buffered formalin (Sigma-Aldrich) and embedded in paraffin. 5 µm thick sections were stained with H&E or ZN. Microscopic images were captured on a Hamamatsu NanoZoomer 2.0 RS slide scanner and its viewing platform (NDP.View2).

Immune cells characterization. Immune cells were isolated from the lungs of 5-6 uninfected or *Mtb* infected mice per group at each time point. The mouse lung was perfused with 5 mL DPBS via the right ventricle of heart following euthanasia and thoracotomy to remove blood from the pulmonary circulation. To obtain a single cell suspension, the mouse lung was sliced into small pieces in RPMI1640 media containing 0.5 mg/mL Collagenase D (Sigma-Aldrich) and 40 units/mL DNAse I (Roche) and incubated at 37°C for 45 min with gentle shaking. Cell suspensions and dissociated tissue were then filtered through a 70 µM cell strainer. After washing the strainer with 4 mL of RPMI1640 media, the cells were pelleted at 500 x g for 5 min at 4°C and washed twice with DPBS. These cells were stained with a live/dead dye (near-

IR fluorescent reactive dye) to determine their viability (1 µL dye in 1 mL cell suspension in DPBS), followed by a single wash. Further, cells were then divided into two groups to identify immune cells with specific surface markers using fluorescence conjugated antibodies specific for (i) myeloid cells (CD45, CD11b, CD11c, siglec F and Gr-1) and (ii) lymphoid cells (CD45, CD3, CD4, CD8, CD25, CD62L and CD44) (Table S1). Antibody cocktails for the two cell types were prepared in BD Horizon Brilliant™ stain buffer (BD Biosciences). Cells were incubated with these cocktails on ice for 20 min followed by a wash with DPBS containing 3% FBS and fixed with fixation and permeabilization solution (BD Biosciences). For FoxP3 and IFN-γ detection, cells were cultured in RPMI-1640 containing 5 ng/mL PMA, 500 ng/mL Ionomycin and 10 µg/mL brefeldin A (Sigma-Aldrich) at 37°C for 4 hours. Cells were then harvested, stained with a live/dead dye and surface stained with antibodies specific for CD45, CD3, CD4, CD8 and CD25 on ice for 20 min followed by two DPBS washes. Cells were fixed and permeabilized using the eBioscience™ Foxp3/Transcription Factor Staining Buffer Set (ThermoFischer) and then stained with antibodies specific for FoxP3 and IFN-γ. Cells were washed twice with DPBS before analysis. Flow cytometry acquisitions and analyses were carried out using a FACS LSRFortessa™ with FACSDiva software (BD Biosciences, San Jose, CA). Data were further analyzed using FlowJo™ v10.4.2 (Tree Star, Ashland, OR).

Serum cytokines measurements. To measure serum cytokines, serum was isolated from age matched uninfected CSE$^{-/-}$ and WT mice, and at 2- and 3-weeks post-infection and stored at -80°C until further analysis. Serum was diluted 1:4 and cytokine levels were determined using the magnetic bead-based Bio-Plex Pro Mouse cytokine 23-Plex, Group I (Bio-Rad), as per manufacturer instructions and measured the cytokines using Bio-Plex 200 instrument. The Bio-Plex Manager software was used to determine the concentrations of 23 cytokines in pg/mL in each sample from the measured median fluorescence intensity (MFI) and the standard curves of the cytokines. Four-six mice were used per group for each timepoint.

Preparation of peritoneal macrophages and *Mtb* infection. $CSE^{-/-}$ and WT mice were injected intraperitoneally with 2 mL of 4% thioglycollate (Brewer modified, BBL, BD Biosciences). Five days later, peritoneal exudate cells were collected from the peritoneal cavity by injecting ice-cold DMEM medium into the peritoneal cavity. Cells were cultured overnight at 37°C, 5% CO_2 in DMEM medium supplemented with 10% fetal bovine serum (FBS, Thermo Scientific HyClone) and penicillin-streptomycin (100 U/mL). The following day, the adherent peritoneal macrophages were washed with the same media to remove nonadherent cells. For further studies, the peritoneal macrophages were cultured in the same medium without antibiotics. Peritoneal macrophages were infected with *Mtb* at a MOI of 4 for the extracellular flux analysis, metabolite analysis, flow cytometry analysis and cytokine analysis and at a MOI of 1 for the CFU determination.

Western blot. Uninfected and *Mtb* infected mouse lungs or peritoneal macrophages isolated from $CSE^{-/-}$ and WT mice were homogenized or lysed, respectively, in RIPA buffer containing protease inhibitors (Complete Tablets, Roche). Lungs were homogenized in Dounce homogenizer, while macrophages were lysed by one freeze-thaw cycle and passing the lysate through a 26 G needle. After spinning the lysate at 15000 x g, the protein supernatant was quantified using the BCA Protein Assay kit (Thermo Fischer Scientific). 50 µg of lysate was resolved on a 10% SDS-PAGE (Bio-Rad) and transferred to a buffer-soaked PVDF membrane (Bio-Rad) using Trans-Blot Turbo transfer system (Bio-Rad). Membranes were blocked in 5% fat-free dried milk in 1 x TBS (Tris Buffered saline?) containing 0.1% Tween 20 (TBST) for 1 hour. After blocking, membranes were incubated with primary antibodies against CSE (ProteinTech,1:3000), CBS (Novus Biologicals, 1:2000) and MPST (Novus Biologicals, 1:2000) diluted in 5% milk in TBST for 1.5 hours (CBS and MPST) or overnight at $4^{o}C$ (CSE) followed by HRP conjugated anti-rabbit IgG (R&D Systems). Protein bands were detected by peroxidase activity using ECL substrate (Clarity Western ECL Substrate or SuperSignal West Femto Maximum Sensitivity Substrate) and images were captured on a ChemiDoc™ MP (Bio-Rad).

RNA preparation and RT-qPCR. RNA was extracted from uninfected and *Mtb* infected macrophages isolated from $CSE^{-/-}$ and WT mice using Aurum Total RNA mini kit (Bio-Rad) as per manufacturer's instructions. Total RNA was extracted from 6 replicates per group (1 mouse per replicate). RNA integrity (RNA Quality Indicator (RQI) >9.5) and quantity (ng/uL) were measured using Experion RNA StdSens Starter kit (Bio-Rad) in Experion automated electrophoresis system (Bio-Rad). cDNA was generated from 500 ng of total RNA using the iScript Advanced cDNA synthesis Kit (Bio-Rad). Quantitative real-time PCR was performed using SsoAdvanced Universal SYBR Green Supermix (Bio-Rad) on CFX96 Touch Real-Time system (Bio-Rad) and analysed using CFX Manager version 3. The relative gene expression was normalized to the mouse β-actin gene as an internal control.

Metabolites and amino acid detection with LC-MS/MS. Mouse peritoneal macrophages were plated at 1.5 x 10^6 cells per well in 6-well plate, infected with *Mtb* at a MOI 4.0 per well and incubated at 37°C for 24 hrs. Infected cells were left untreated or treated with indicated concentrations of GYY4137. After 24 hours, the cells were washed twice with DPBS (Lonza Bioscience), prior to adding ice-cold methanol:water (1:1) to the macrophages to quench the metabolites. The cells were then incubated in dry ice for 5 minutes. Cells were lysed by bead beating in a Roche MagNA Lyser at 7,000 rpm for 1 min, repeated for two cycles with 5 min of cooling on ice between the cycles. The lysed cells were centrifuged at a 18 000 x g at 4°C and a 50 uL aliquot of the supernatant was used for protein estimation. Supernatants were spun through a 0.22 µm filter and the filtrate was evaporated using a speed vacuum concentrator at 42°C. The dried pellet was reconstituted in 150 µL water and filtered through a 0.22 µm spin filter for LC-MS/MS analysis (2). For amino acid quantitation, 50 µL of the metabolite extraction was diluted with 50 µL acetonitrile before LC-MS/MS analysis (2). Protein content of the lysed cells was estimated with the Micro BCA™ Protein Assay Kit (Thermo Fisher Scientific) to normalize the area under the curve (AUC) of metabolites and

amino acids per mg of protein. Standards of all the individual metabolites and amino acids (purchased from Sigma-Aldrich) were used as positive controls for LC-MS/MS detection.

H_2S measurement. H_2S was measured in the culture supernatant of peritoneal macrophages isolated from $CSE^{-/-}$ and the WT mice using the method described previously (3) with some modifications. Uninfected and *Mtb* infected macrophages 20 hours post-infection were incubated with the following treatments: (i) untreated, (ii) 2 mM cysteine, (iii) 1.0 mM PAG followed by 2 mM cysteine and (iv) 3.0 mM PAG followed by 2 mM cysteine. For PAG treatment followed by cysteine treatment, cells were treated first with PAG for the last 20 min of the 20 hours infection and then cysteine was added without changing the media. Then the cells were incubated for a further 4 hours, followed by H_2S measurement.

Two methods were used to detect H_2S: (i) Methylene blue method was used to determine total sulfide concentration, $[S^{2-}_{total}] = [H_2S] + [HS^-] + [S^{2-}]$. Here, 1.0 mL of culture supernatant was slowly added to 133 µL of 20 mM N,N-dimethyl-p-phenylenediamine sulfate in 7.2 M HCl followed by the addition of 133 µL of 30 mM iron(III)chloride ($FeCl_3.6H_2O$) in 1.2 M HCl to the bottom of the mixture in the tube. The mixture was left for 15 min without further mixing. Then, 200 µL of the mixture was placed in 96-well clear plate and the development of blue colour was measured in the spectrophotometer at 670 nm. The concentration of H_2S was determined using a standard curve from 0 to 100 µM (R^2 = 0.9998) of NaHS prepared from a freshly prepared 1 mM stock in deoxygenated water. (ii) H_2S was also measured with a Unisense H_2S microsensor as per manufacturers guidelines (Unisense, Denmark). These microsensors (H_2S-500 and SULF-500) are specific for H_2S detection with a linear range of H_2S detection from 0 to 300 µM. Freshly prepared NaHS standards from 0 to 50 µM were used to plot a standard curve (R^2 = 9987) to determine the H_2S concentrations in the samples. Four biological replicates were used to measure H_2S; macrophages for each replicate were pooled from two mice, hence a total of eight mice of each group (uninfected or infected $CSE^{-/-}$ or WT) were used.

***In vitro* CFU assay.** Peritoneal macrophages from CSE$^{-/-}$ and WT mice were plated at 1.5 x 10^6 cells per well in 6-well plate. Cells were infected with *Mtb* at a MOI of 1 and incubated at 37°C for 4 hours. Cells were washed twice to remove non-phagocytosed bacilli. These infected cells were left untreated or treated with either 50 µM or 200 µM GYY4137 (a slow releaser of H_2S) (50 µM and 200 µM) or 1.0 mM or 4.0 mM PAG (DL-Propargylglycine, a specific inhibitor of CSE). As an experimental control for GYY4137, cells were treated with spent (decomposed) 200 µM GYY4137 that was previously aerated for at least 120 days. At 0, 2 and 4 days, cells were lysed with DPBS containing 0.05% SDS and CFUs were determined by plating serial dilutions of lysates on 7H11 agar plates supplemented with 10% OADC. Plates were incubated at 37°C with 5% CO_2 for 4 weeks to determine CFU counts.

***In vitro* cytokine detection.** Peritoneal macrophages from CSE-/- and WT mice were plated at 1.5 x 10^6 cells per well in 6-well plate. Cells were infected with *Mtb* at a MOI 4.0 per well and incubated at 37 ^{o}C. After infection, cells were left untreated or treated with 50 µM or 200 µM GYY4137. Culture supernatants were collected after 24 hours, centrifuged at 500 x g for 5 min and filtered through a 0.22 µm filter column. The cytokines IL-1β, IL-6, IL-8 (CXCL-8), TNF-α and IL-10 were quantified in the supernatant via a cytometric bead array (CBA) using a FACS LSRFortessa™ and FACSDiva software (all BD Biosciences) according to the manufacturer's instructions.

RNA sequencing and analysis. Total RNA (RQI>9.5) extracted from *Mtb* infected macrophages isolated from CSE$^{-/-}$ and WT mice were submitted for sequencing at the Heflin Genomics Core Facility (University of Alabama at Birmingham). RNA sequencing was performed using Illumina NextSeq500 as per manufacturer's protocol (Illumina). The mRNA library was prepared using the SureSelect Strand Specific mRNA library kit as per manufacturer's protocol (Agilent). To construct the library, mRNA was purified by two rounds

of polyA selection using oligo dT-containing magnetic beads. After purification, mRNA was randomly fragmented and then subjected to cDNA first strand synthesis using reverse transcriptase and random primers with inclusion of Actinomycin D (2.4 ng/μL). Second strand cDNA synthesis was performed using DNA polymerase I and RNaseH. cDNA was purified using AMPure XP beads and the ends of the resulting cDNA were blunted, A-tailed and ligated to adaptors for indexing to allow for multiplex sequencing. cDNA libraries were quantitated using qPCR (Roche, LightCycler 480) with the Kapa Biosystems kit for Illumina library quantitation (Kapa Biosystems). Then, clusters were generated according to manufacturer's recommendations for onboard clustering (Illumina). Paired-end 75-bp read length was used for better alignment to the reference genome. Sequenced sample reads were subjected to quality checks using MultiQC (4) to ensure PHRED >30. STAR (version 2.5.3a) was used to align the RNA-Seq fastq reads and annotated to the *Mus musculus* reference genome (GRCm38.p6, Release M18) from Gencode (5). HTSeq-count version 0.9.1 was then used to calculate transcript abundances (raw counts) or number of reads mapped to each gene. DESeq2 (version 1.18.1) was applied to the count files for normalization and differential expression of the gene (6) within R (version 3.4.2). Heatmaps were generated using the pheatmap package (version 1.0.12) (7) in the R statistical software (version 3.6.0) (R Foundation for Statistical Computing, Vienna, Austria., 2019. https://www.R-project.org/). Gene expression values were scaled by row and were arranged by hierarchical clustering. Gene lists for specific metabolic pathways were downloaded from the Kyoto Encyclopedia of Genes and Genomes (https://www.genome.jp/kegg/pathway.html)(8), and gene lists for electron transport chain complexes were obtained from the Mouse Genome Database (http://www.informatics.jax.org/)(9). Raw sequence reads were uploaded to NCBI Gene Expression Omnibus (GSE143619).

Extracellular flux analysis. The mitochondrial and glycolytic functions of the peritoneal macrophages were measured using an Agilent Seahorse extracellular flux analyzer (XF96) which measures the oxygen consumption rate (OCR) and the extracellular acidification rate

(ECAR) of the cells. Mouse peritoneal macrophages were seeded at 65000 cells per well into a XF96 cell culture microplate. After 12 hours, cells were infected with *Mtb* at a MOI of 4 for 4 hours and then treated with GYY4137 (50 µM) and PAG (1 mM) for 20 hours. An XF Cell Mito Stress Test (CMST) was used to calculate the respiratory parameters of the peritoneal macrophages using modulators of the electron transport chain (ETC). Optimized concentrations of these modulators were: 1.5 µM for oligomycin (complex V inhibitor) (Sigma Aldrich), 1.5 µM for carbonilcyanide *p*-triflouromethoxyphenylhydrazone (FCCP, ETC uncoupling agent) (Sigma-Aldrich) and 0.5 µM for antimycin A/rotenone (complex III/I inhibitor) (Sigma Aldrich). To determine the glycolytic function of the cells, a Glycolysis Stress Test (GST) was performed using glucose (10 mM), oligomycin (1.5 µM) and 2-deoxyglucose (50 mM) (Sigma-Aldrich). After infection and treatment of the peritoneal macrophages, cell media was exchanged for XF assay media (DMEM supplemented with 1 mM sodium pyruvate and 2 mM GlutaMax (Thermo Fisher) and 0- or 25-mM glucose for GST and CMST assay media, respectively). Cells were incubated for 1 hour at 37˚C in a non-CO_2 incubator prior to XF analysis. Drugs were prepared at 10x their required concentration and loaded into their respective drug ports in the XF cartridge. All assay media was pre-warmed to 37 ˚C and pH corrected to 7.4 (CMST) and 7.35 (GST).

Mitochondrial membrane potential and ROI & RNI. Mouse macrophages were plated at 1.5×10^6 cells per well in a 6-well plate and infected with *Mtb* at a MOI 4.0. After 24 hours of incubation, cells were harvested by scraping and centrifuged at 500 *x g* for 5 min. The cell pellet was resuspended in 200 µL DMEM media supplemented with 10% FBS containing 50 nM MitoTracker Green FM (excitation/emission 490/516 nm) and 25 nM MitoTracker Deep Red FM (excitation/emission 644/665 nm) (Molecular Probes). The cells were incubated at 37°C for 15 min, followed by two washes with DMEM without FBS. The cells were then resuspended in 200 µL of Hank's balanced salt solution with calcium and magnesium containing 5 µM MitoSOX™ Red (excitation/emission 544/580 nm) (Molecular Probes) and incubated at 37°C for 15 min, followed by one wash. Data acquisition was performed on a BD

FACSAria^{TM} III flow cytometer and analysed using FACSDiva software (all BD Biosciences) and FlowJo^{TM} v10.4.2 (Tree Star, Ashland, OR).

Statistics. All experiments were performed on 3 to 6 biological replicates and the data were expressed as mean ± SD or mean ± SEM (for serum cytokine data). Statistical significance of the data was determined using GraphPad Prism software (Version 7.0c), (GraphPad Software, Inc.). Specific statistical tests appear in the figure legends and include the Student's unpaired t-test (two-tailed), one-way or two-way ANOVA.

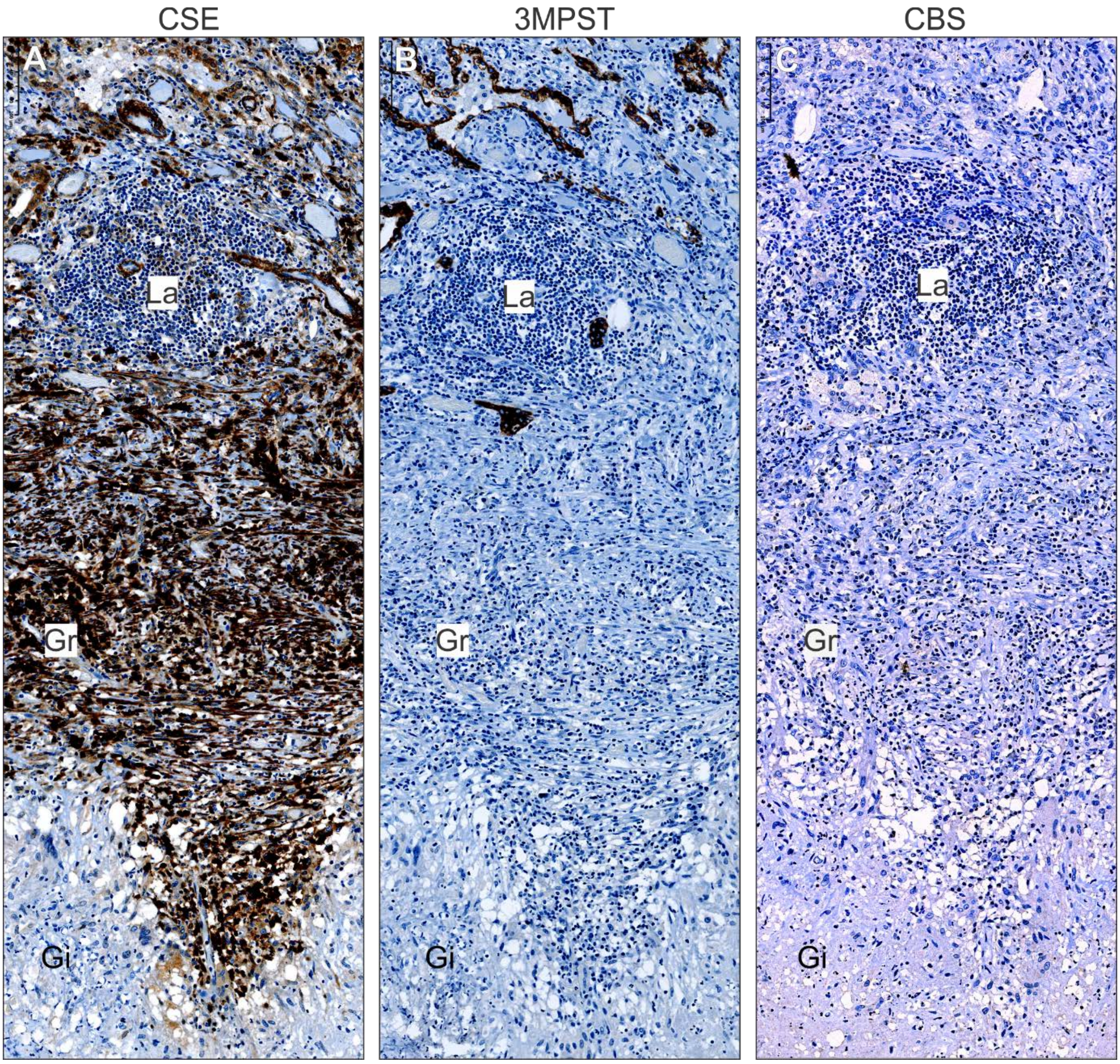

Figure S1. IHC staining of CSE, MPST and CBS in the cavity wall of human TB lung tissue. Medium power magnification of CSE (**A**), MPST (**B**) and CBS (**C**) staining. Note the absence of staining in the granulomatous inflammatory layer (Gi), but strong staining of CSE in the granulation layer (Gr). CSE weakly stained cells in the lymphoid aggregate whereas CBS stained negative for all cells. Gi; granulomatous inflammation layer, Gr; granulation layer, La; lymphoid aggregate.

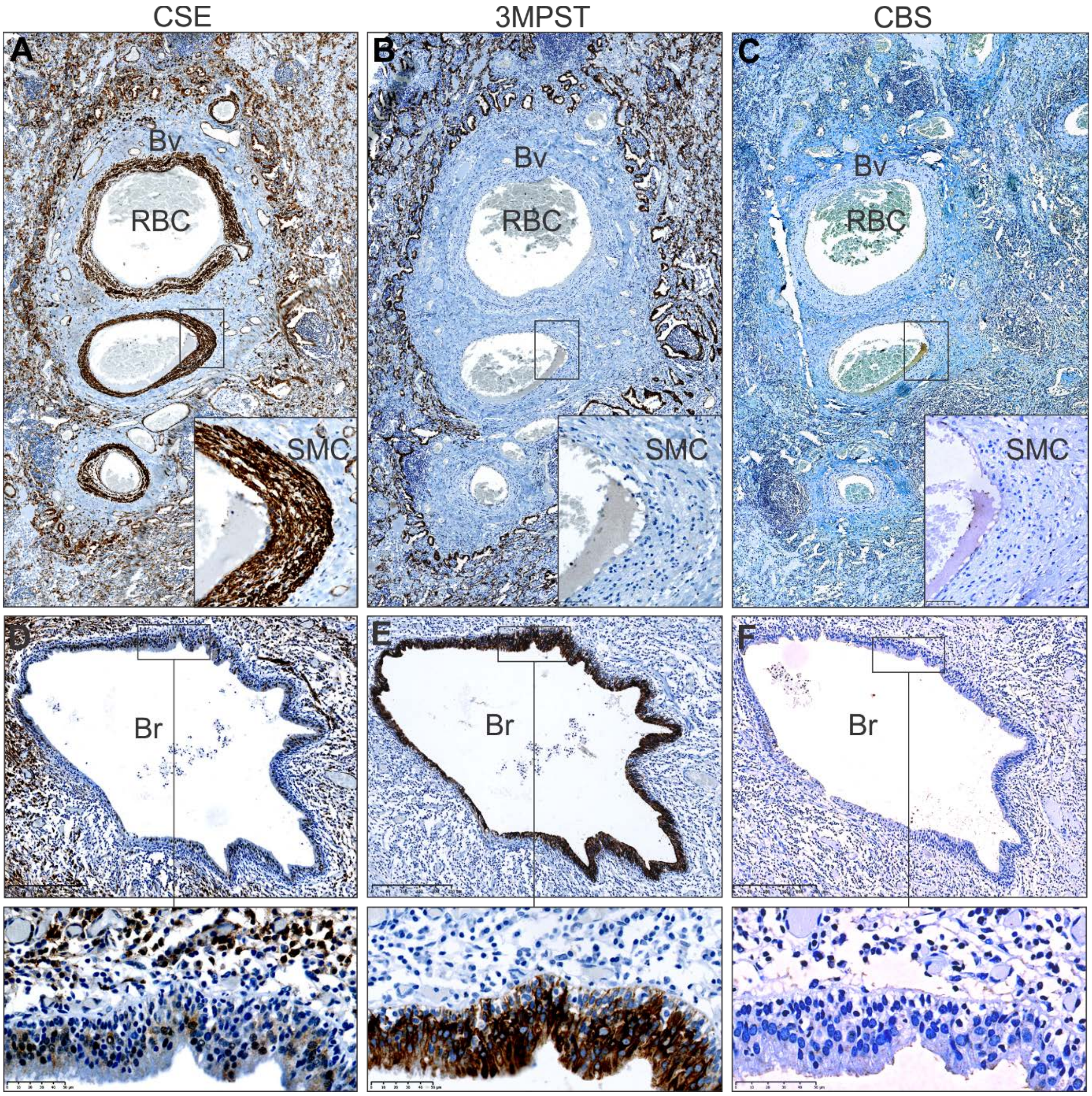

Figure S2. IHC staining of CSE, MPST and CBS in blood vessels and bronchi of human TB lung tissue. Shown is bright staining of CSE in smooth muscle cells (**A**) (see insets for high power images) whereas MPST (**B**) and CBS (**C**) stained negative. CSE stained weakly positive in bronchial epithelial cells (**D**) whereas MPST stained strongly positive in these cells (**E**). CBS stained negative for bronchial epithelial cells (**F**). RBC; red blood cells, Bv; blood vessels, Br; bronchus, SMC; smooth muscle cells

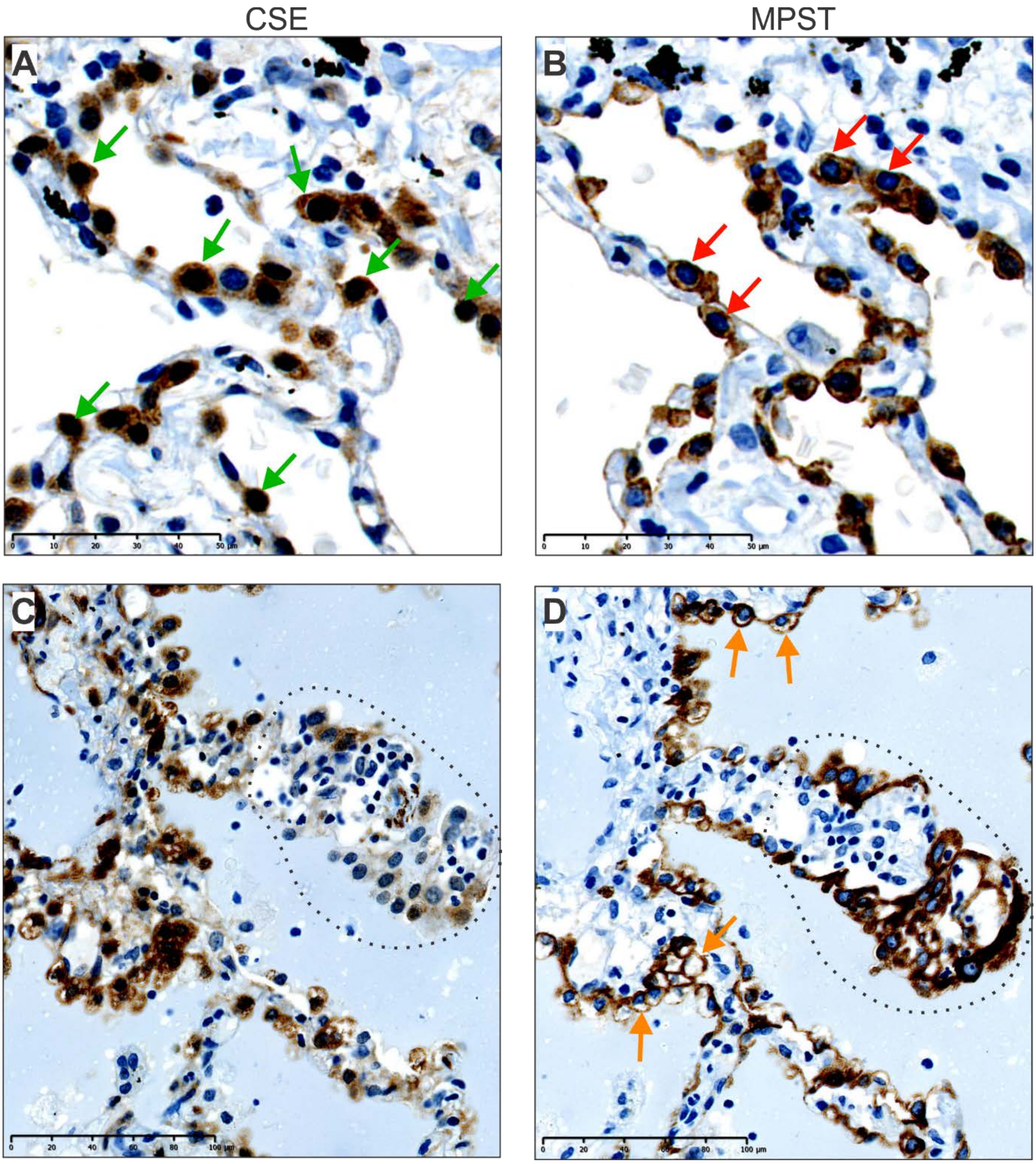

Figure S3. IHC staining of CSE and MPST in the adjacent lung of human TB lung tissue. High power magnification of CSE (**A, C**) and MPST (**B, D**) staining of uninvolved human TB lung tissue. CSE and MPST brightly stain alveolar pneumocytes (**A**; green arrows and **B**; red arrows). Note the CSE and MPST co-staining of pneumocytes in **C** and **D** (dotted circle). CSE stains the nuclei and cytoplasm of most cells brightly (**A**; green arrows). MPST stains the membranes (**D**; orange arrows) and cytoplasm (**B**; red arrows) of cells brightly (**B, D)**.

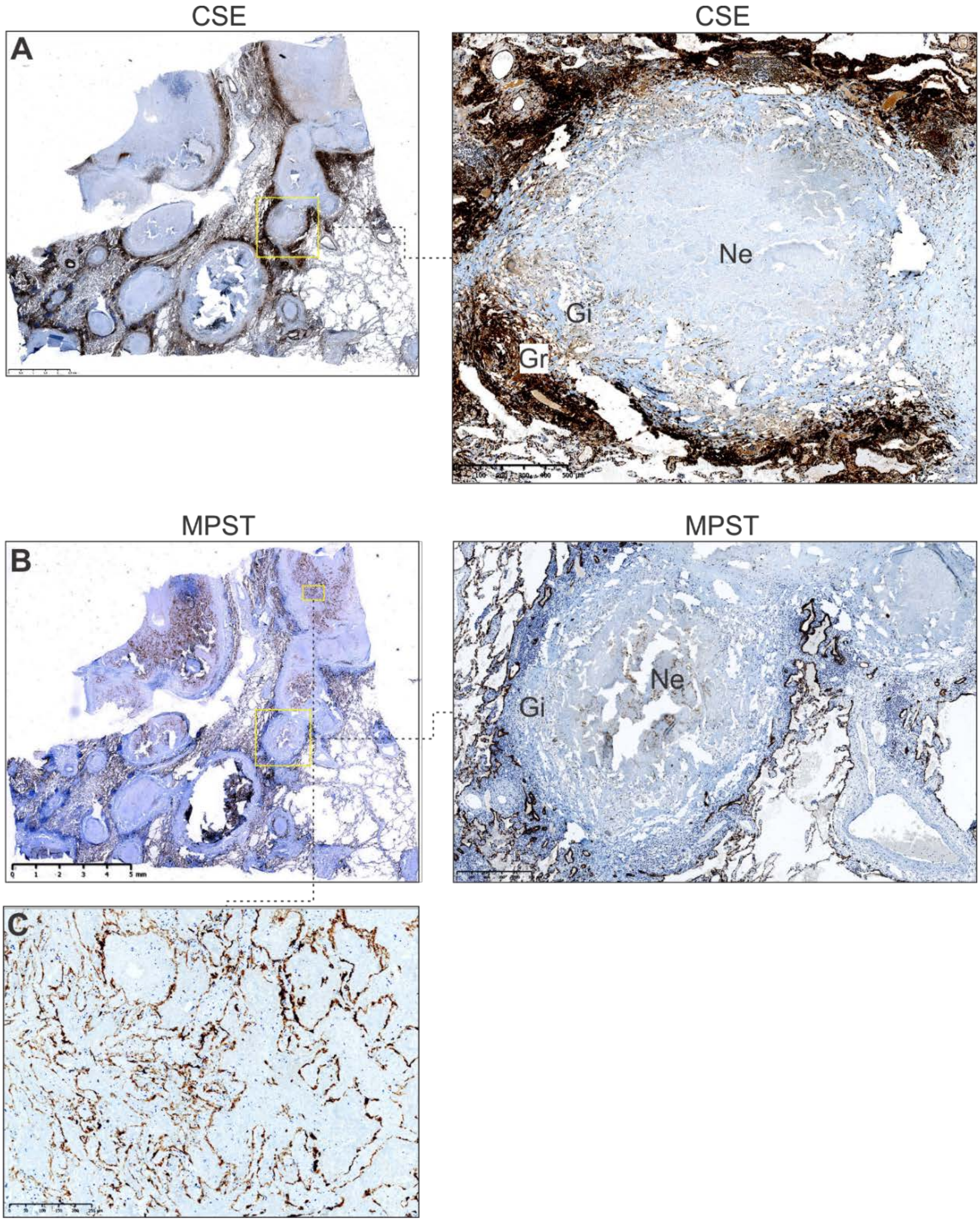

Figure S4. **IHC staining of CSE and MPST in a necrotic granuloma within human TB lung tissue.** Low (**A**) and medium power magnification of the yellow box of necrotic tubercles stained with CSE. Note the bright positive staining of CSE in the granulation layer and absence of staining in the granulomatous inflammation layer (medium power inset). (**B**) Low and medium power (inset) magnification of necrotic tubercles stained with MPST. Note the bright IHC staining of MPST in epithelial cells surrounding the tubercle and lack of staining in the granulomatous inflammation layer. (**C**) Medium power magnification of MPST positive staining of remnants of alveoli within the tubercle. Ne; necrosis, Gi; granulomatous inflammation layer, Gr; granulation layer

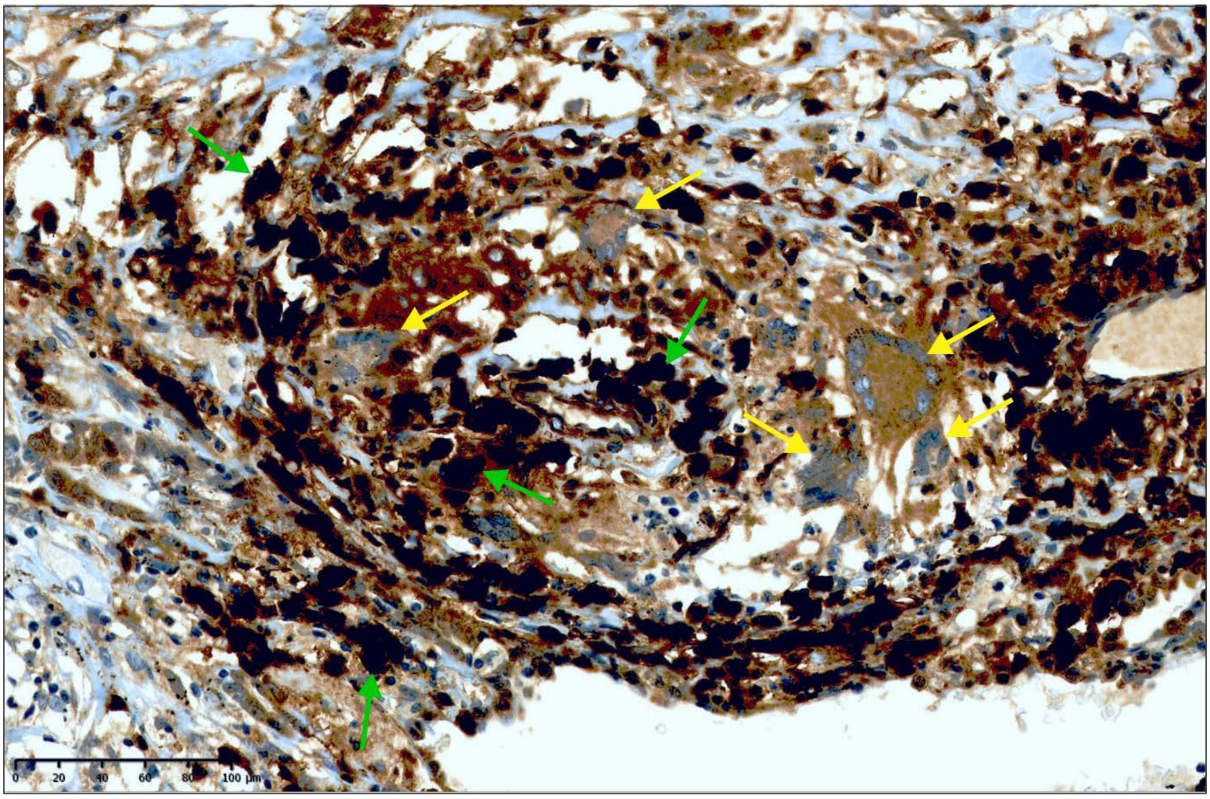

Figure S5. IHC staining of CSE in a non-necrotic human TB granuloma. High power magnification of giant cells (yellow arrows) stained positive for CSE; note the brightly stained histiocytes (green arrows) within and surrounding the granuloma.

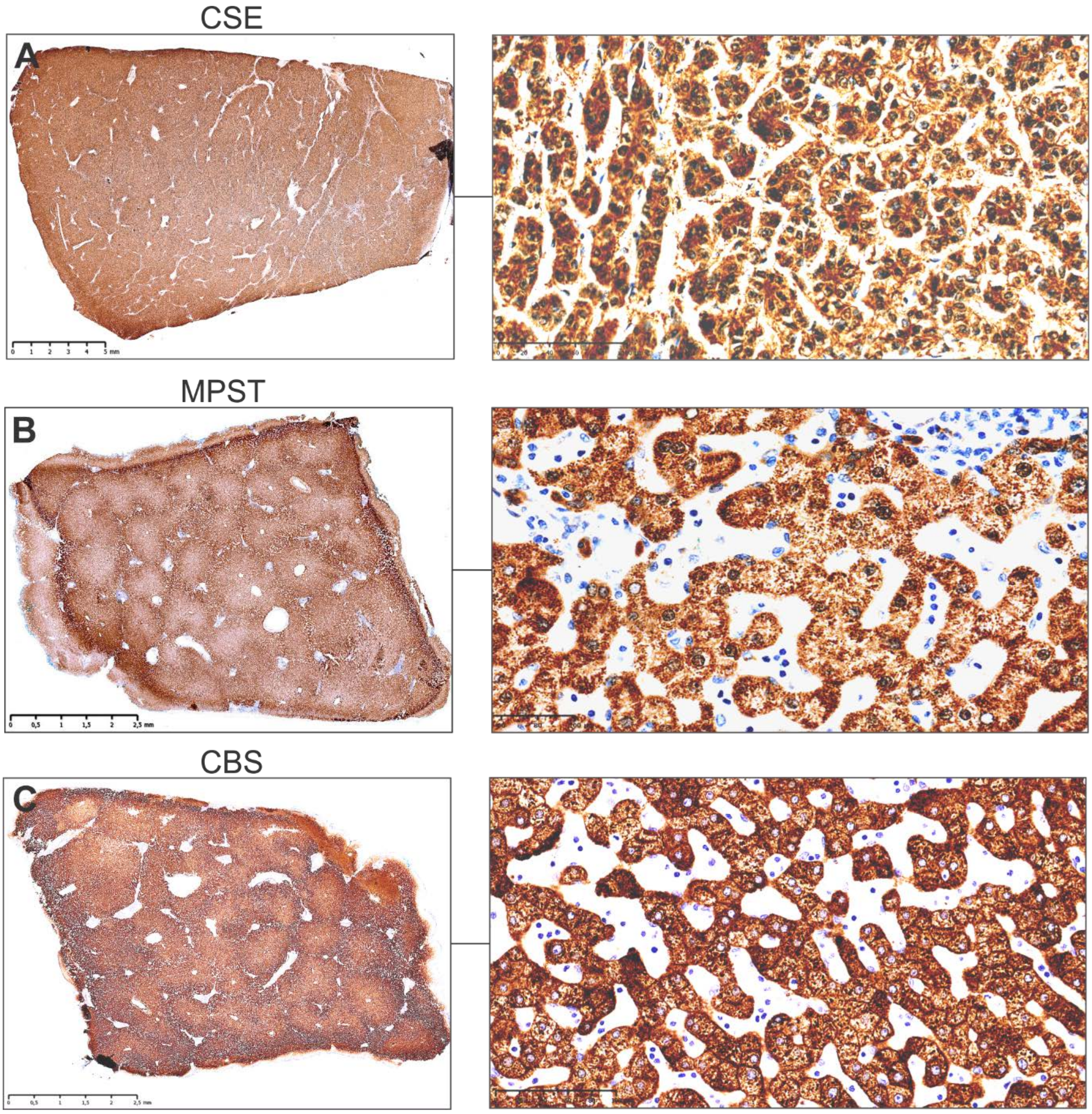

Figure S6. IHC staining of CSE, MPST and CBS in human liver control tissue. Low and medium power images of liver tissue stained positive for CSE (**A**), MPST (**B**) and CBS (**C**).

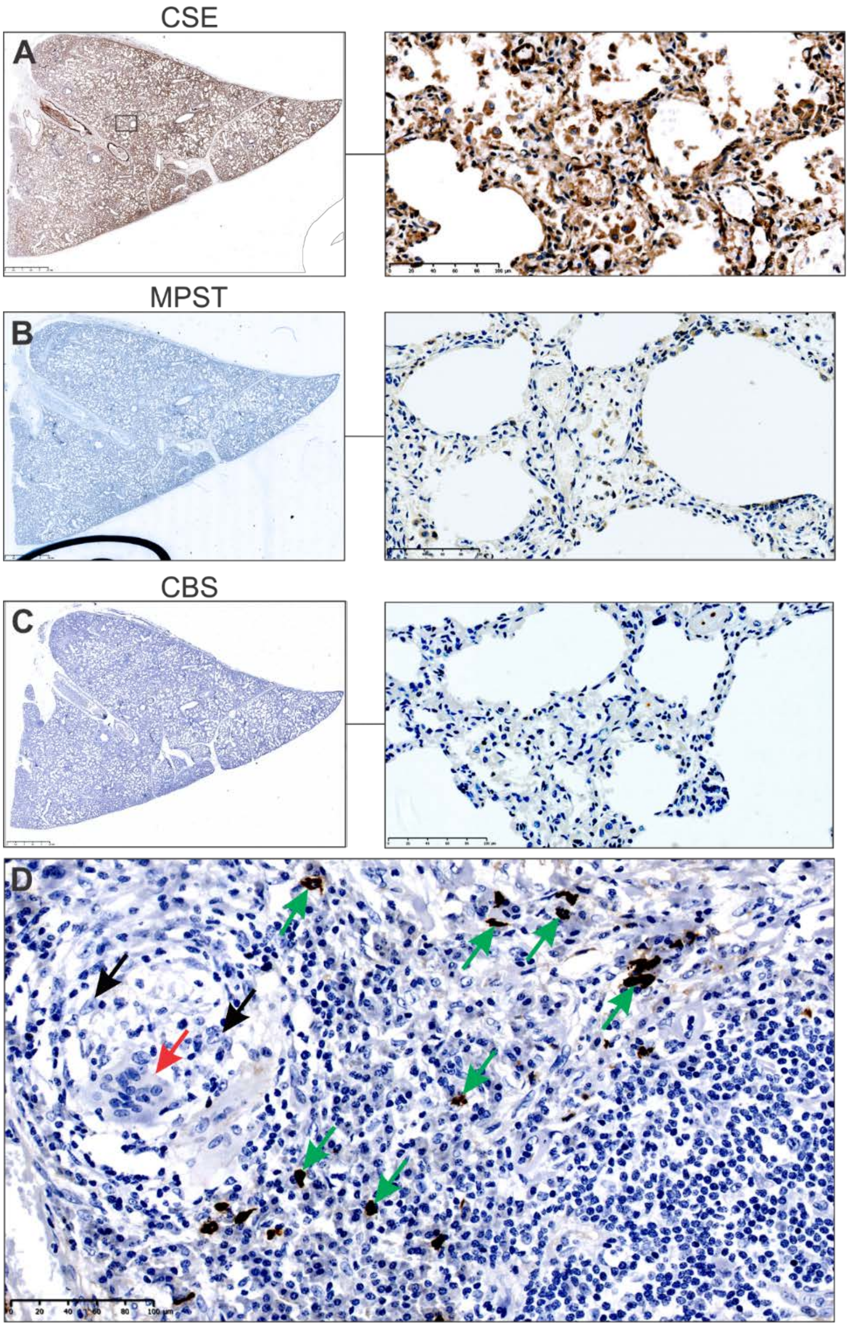

Figure S7. IHC staining of CSE, MPST and CBS in healthy human lung tissue and isotype control staining. Low and medium power images of lung tissue stained positive for CSE (**A** and medium power inset), MPST (**B** and medium power inset), but negative for CBS (**C** and medium power inset). (**D**) Immunonegative rabbit IgG isotype control. High power demonstration of granuloma confirming immunonegative epithelioid histiocytes (black arrows) and Langhans giant cell (red arrow). Note the IgG4 immunopositive plasma cells (green arrows) serving as a positive, in-built isotype control response, and surrounding stromal immunonegative mesenchymal and lymphoid cells. In contrast, the viable intra-alveolar and interstitial cellular components were immunonegative with clear cytoplasm's and hematoxyphilic (blue) nuclei because of hematoxylin counterstaining.

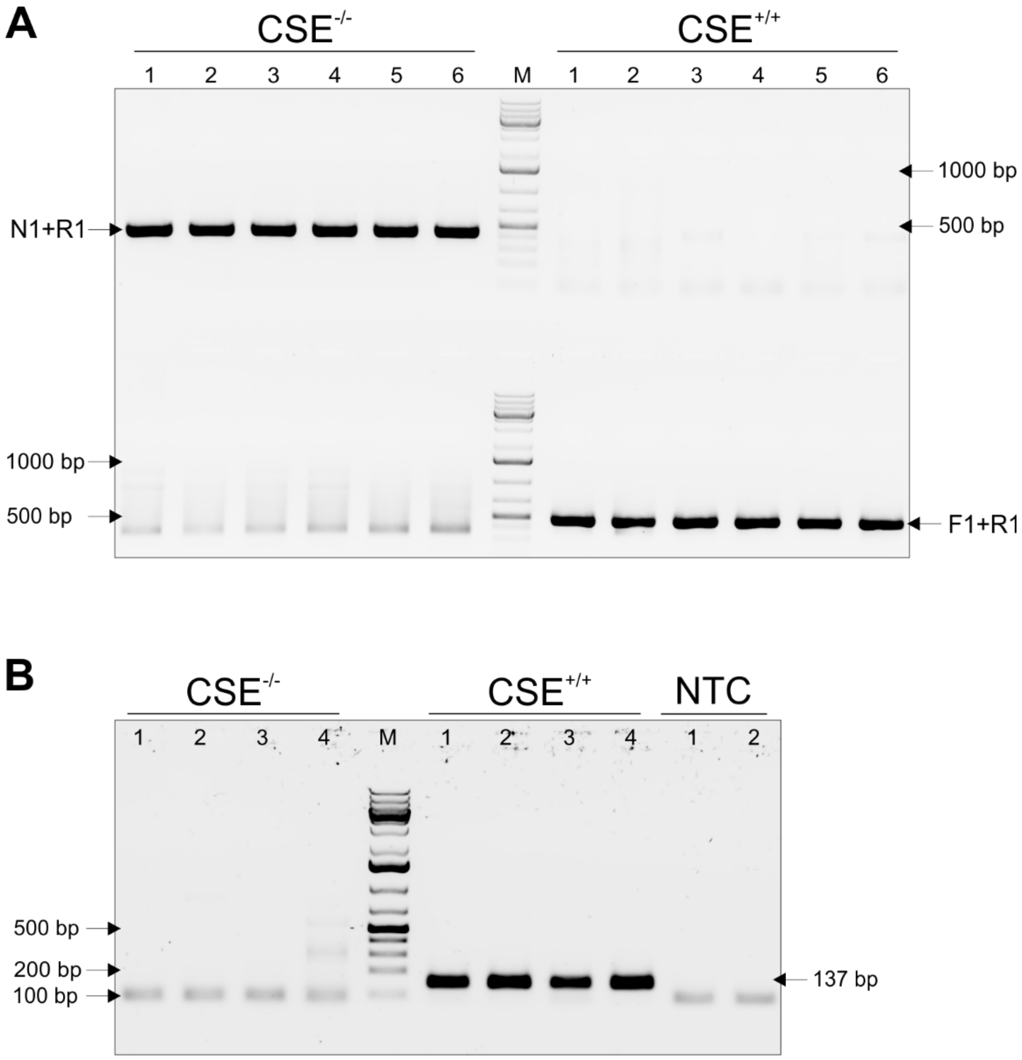

Figure S8. Genotyping of CSE$^{-/-}$ and CSE$^{+/+}$ (WT) mice. Genotyping was performed on genomic DNA isolated from the blood using PCR with three primer sets (F1, N1 and R1). (**A**) PCR amplification in the N1+R1 PCR reaction and absent in F1+R1 PCR reaction confirms *CSE* deletion in mice, whereas amplification in the F1+R1 PCR reaction and absent in the N1+R1 PCR reaction confirms the presence of *CSE* in WT mice. The expected amplification size from each set of primers was between 400 and 500 bp. Genotyping was performed on the blood genomic DNA isolated from six WT and CSE$^{-/-}$ mice each. (**B**) Lack of CSE mRNA expression in CSE$^{-/-}$ mice. PCR amplification of CSE$^{-/-}$ and WT mice cDNA (four mice in each group) was performed to check for CSE expression using gene specific primers CSEqF and CSEqR (listed in Table S1). As a negative control, PCR without cDNA was performed in duplicate (NTC – no template control).

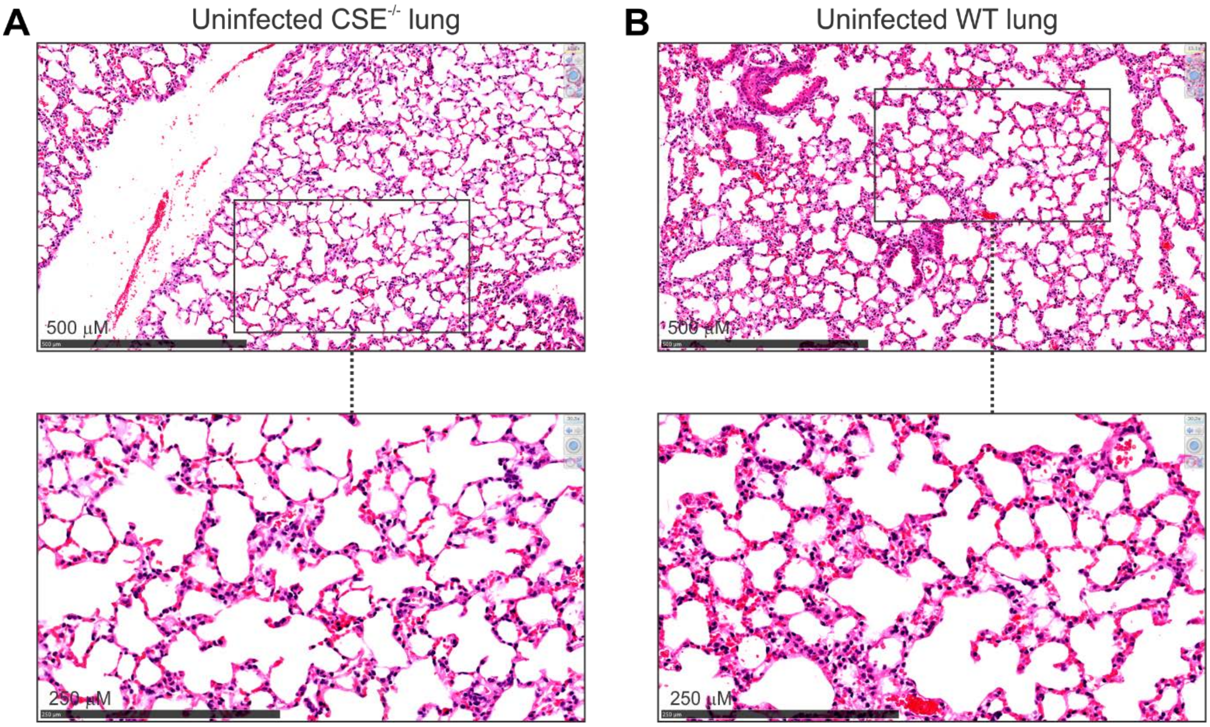

Figure S9. Microscopic view of uninfected CSE$^{-/-}$ and WT mouse lungs. Microscopic views of H&E stained lung sections of uninfected (**A**) CSE$^{-/-}$ and (**B**) WT mouse lungs demonstrating micro-anatomy. Inset, higher magnification.

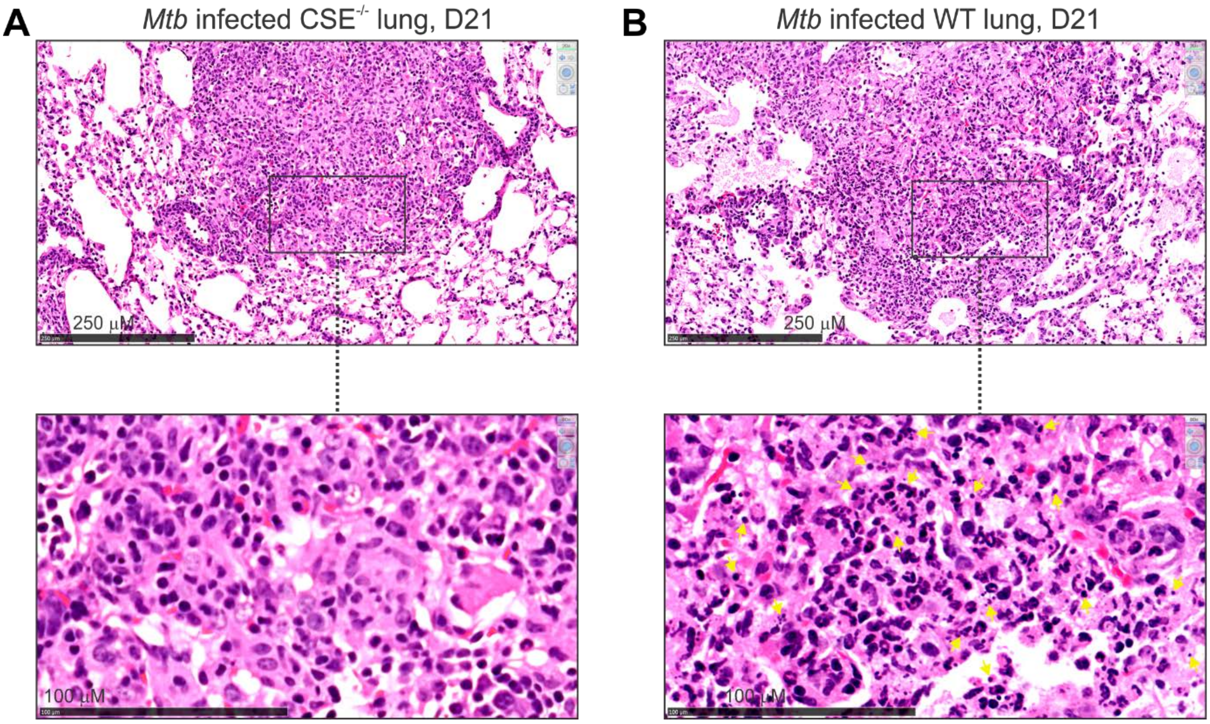

Figure S10. Histopathological differences of *Mtb* infected CSE$^{-/-}$ and WT mouse lungs. Microscopic views of H&E stained lung sections of *Mtb*-infected (**A**) CSE$^{-/-}$ and (**B**) WT mouse lungs at day 21. Inset: Higher magnifications demonstrate karyorrhexis in the infected WT lungs (yellow arrows) that is mostly absent in the infected CSE$^{-/-}$ lungs.

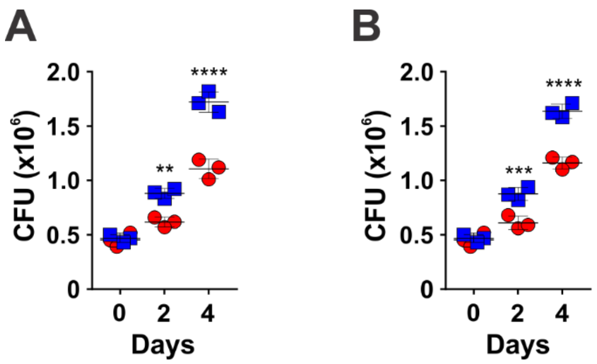

Figure S11. ***In vitro*** **CFU of** ***Mtb*** **infected CSE$^{-/-}$ and WT macrophages treated with spent GYY4137.** Macrophages were (**A**) untreated or (**B**) treated with spent-GYY4137 and infected with Mtb and CFU were determined at indicated time-points. No statistically significant differences were observed in the CFU between the untreated, and spent-GYY4137 treated macrophages.**, p <0.005; ***, p<0.0005; ****, p<0.0001.

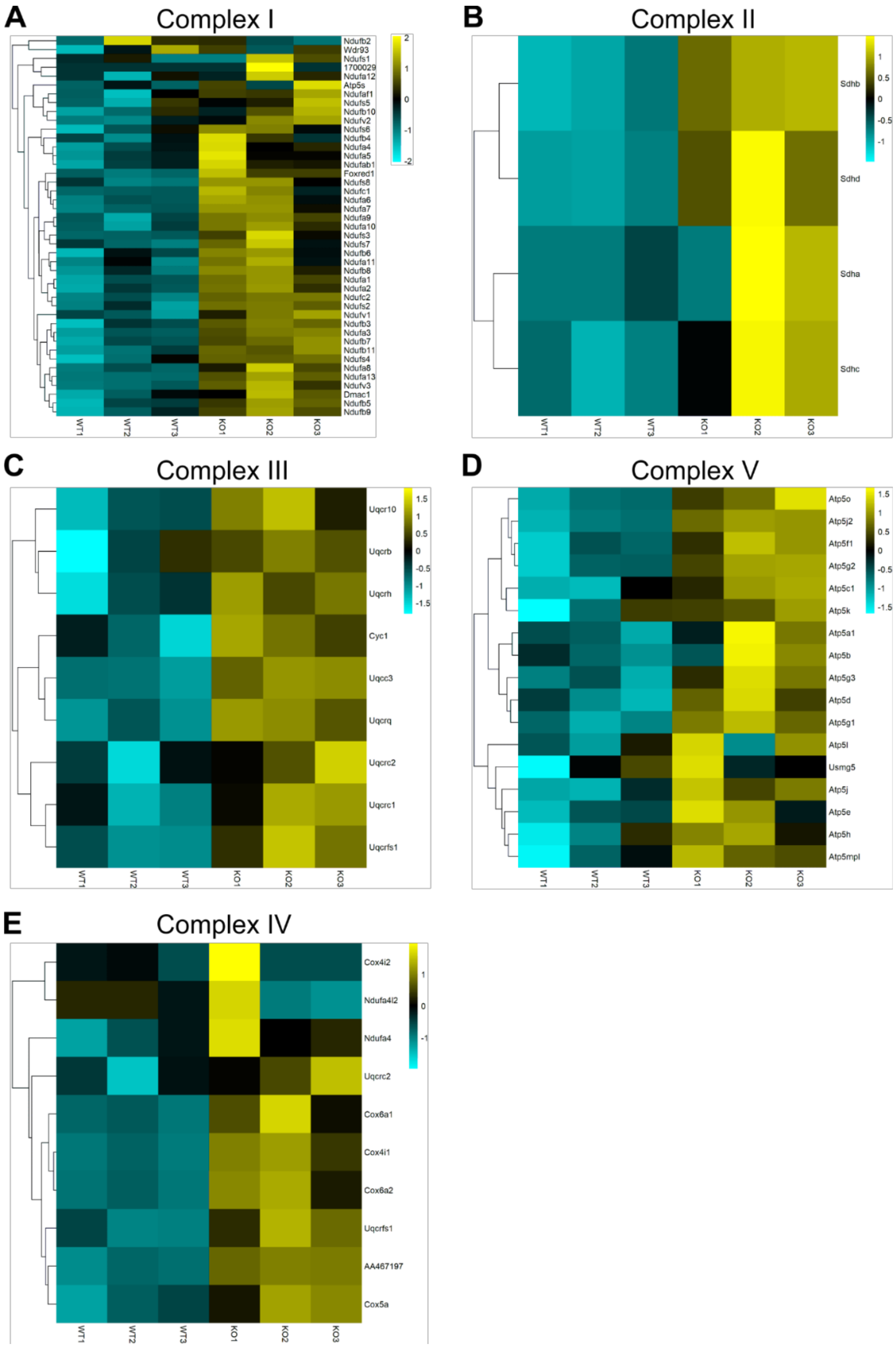

Figure S12. CSE downregulates the gene expression involved in OXPHOS complexes. (**A-E**) Heatmaps generated from RNA-seq data of *Mtb* infected peritoneal macrophages 24 hours post-infection, showing differential expression of genes encoding the subunits of the five complexes involved in OXPHOS: (**A**) Complex I, (**B**) Complex II, (**C**) Complex III, (**D**) Complex IV, and (**E**) Complex V. Peritoneal macrophages were derived from three mice in each group. Data are representative of two independent experiments.

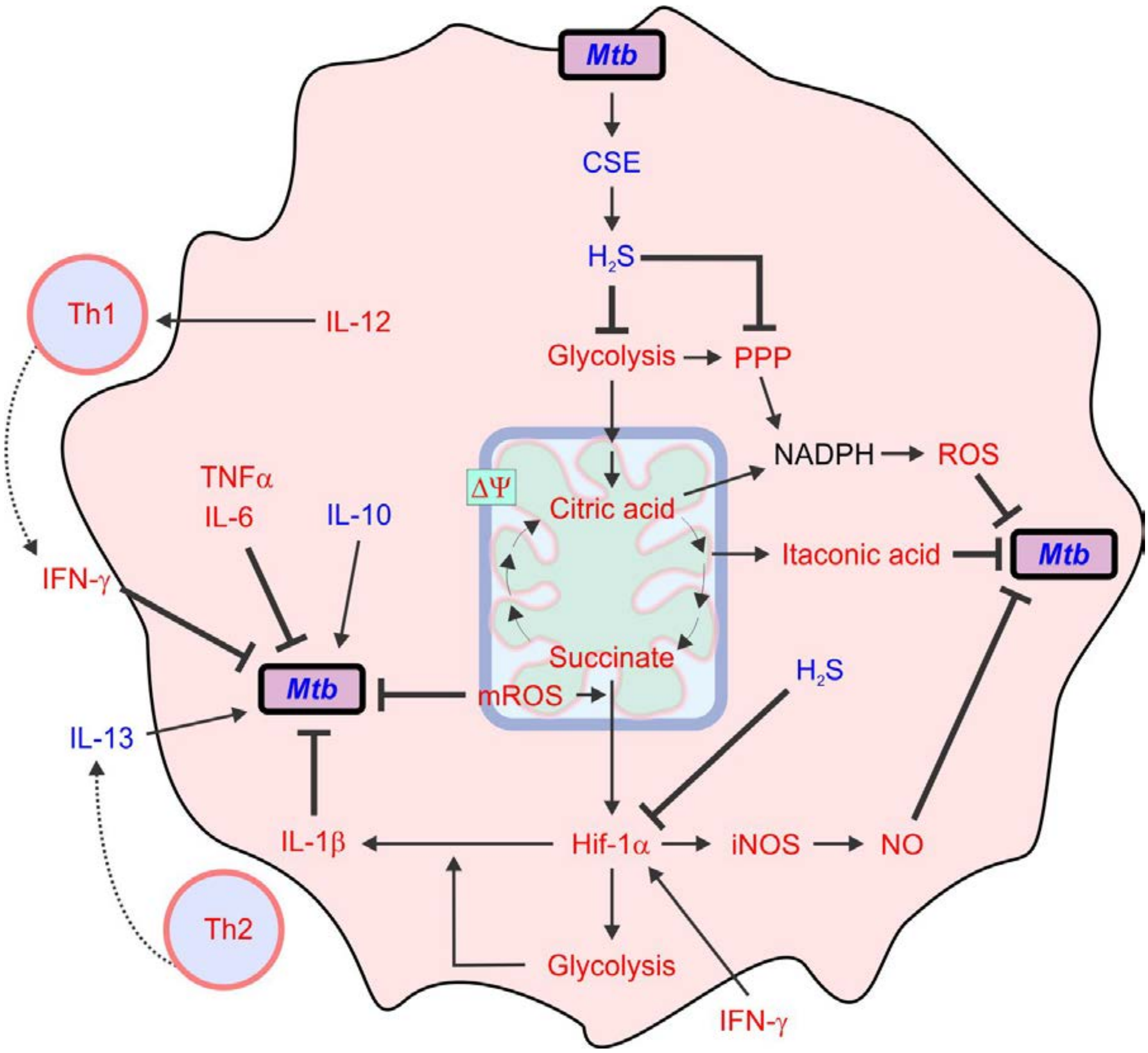

Figure S13. Model depicting the role of CSE and H_2S in TB. *Mtb* regulates proinflammatory and anti-inflammatory mediators of the host by increasing upregulating the production of host CSE generated H_2S. Supraphysiological production of H_2S after *Mtb* infection reduces the levels of glycolysis, PPP and the TCA cycle, in addition to Hif-1α, which regulates the production of inflammatory effectors such as IL-1β, IL-6, TNFα, NO that are essential for the control of *Mtb* proliferation. Blue font indicates increased levels; red font indicates decreased levels.

Table S1: List of reagents used in this study

Antibody	Conjugate	Host	Clone	Catalog	Company
CD45	Alexa Fluor 700	Rat	30-F11	560510	BD biosciences
CD11b	BV605	Rat	M1/70	563015	BD biosciences
CD11c	PerCP-Cy5.5	Hamster	HL3	560584	BD biosciences
Siglec F	PE-CF594	Rat	E50-2440	562757	BD biosciences
Gr-1 (Ly-6G)	BV711	Rat	1A8	563979	BD biosciences
CD3	PE-Cy7	Rat	17A2	560591	BD biosciences
CD4	BV605	Rat	RM4-5	563151	BD biosciences
CD8a	V500	Rat	53-6.7	560776	BD biosciences
CD25	PE-CF594	Rat	PC61	562694	BD biosciences
CD62L	PerCP-Cy5.5	Rat	MEL-14	560513	BD biosciences
CD44	BV650	Rat	IM7	103049	BioLegend
FoxP3	BV421	Rat	MF23	562996	BD biosciences
IFN-γ	BV786	Rat	XMG1.2	563773	BD biosciences
IL-12 (p40/p70)	APC	Rat	C15.6	554480	BD biosciences
CSE	Unconjugated	Rabbit	Polyclonal	12217-1-AP	Protein Tech
CSE	Unconjugated	Rabbit	Polyclonal	NBP1-31759	Novus Biologicals
CBS	Unconjugated	Rabbit	Polyclonal	H00000875-D01P	Novus Biologicals
MPST	Unconjugated	Rabbit	Polyclonal	NBP1-82617	Novus Biologicals
b-actin	Unconjugated	Rabbit	Polyclonal	NB600-503	Novus Biologicals
Hif-1a	Unconjugated	Rabbit	Polyclonal	NB100-479	Novus Biologicals
anti-mouse IgG	HRP	Goat	Polyclonal	NB7539	Novus Biologicals
anti-Rabbit IgG	HRP	Goat	Polyclonal	HAF008	R&D Systems
anti-Rabbit IgG H&L	HRP	Goat	Polyclonal	ab6721	Abcam
Rabbit IgG, Isotype	Unconjugated	Rabbit	Polyclonal	ab37415	Abcam
anti-Rabbit IgG	Alexa Fluor 594	Goat	Polyclonal	8889S	Cell Signal.Tech.
Dye	Description			Catalog	Company
Live/Dead Cell Stain	Near-IR fluorescen, Ex/Em: 750/775 nm			L10119	Molecular Probes
DAF-FM diacetate	Ex/Em: 510/580 nm			D23842	ThermoFisher Sci.
Dihydroethidium	Ex/Em: 510/580 nm			D11347	ThermoFisher Sci.
MitoTracker	Green FM, Ex/Em: 490/516 nm			M7514	Molecular Probes
MitoTracker	Deep Red FM, Ex/Em: 644/665 nm			M22425	Molecular Probes
MitoSOX Red	Ex/Em: 510/580 nm			M36008	Molecular Probes

qRT-PCR primers	Type	Amplicon length	Unique Assay ID	Company
CSE	Intron-spanning	137 bp	CSEqF-CSEqR	Rui Wang(1)
CBS	Intron-spanning	102 bp	qMmuCID0005766	Bio-Rad
MPST	Intron-spanning	169 bp	qMmuCID0009068	Bio-Rad
Hif-1α	Intron-spanning	90 bp	qMmuCID0005501	Bio-Rad
IL-1β	Intron-spanning	95 bp	qMmuCID0005641	Bio-Rad
β-actin	Exonic	109 bp	qMmuCED0027505	Bio-Rad
Cytokines detection kits			Catalog	Company
Bio-Plex Pro™ Mouse Cytokine 23-plex Assay			M60009RDPD	Bio-Rad
BD™ Cytometric Bead Array (CBA)				
IL-1β	CBA Mouse Flex Set		560232	BD biosciences
IL-6	CBA Mouse Flex Set		558301	BD biosciences
IL-8 (KC)	CBA Mouse Flex Set		558340	BD biosciences
TNFα	CBA Mouse Flex Set		558299	BD biosciences
Reagents/Kits			Catalog	Company
N,N-dimethyl-p-phenylenediamine sulfate			186384-25G	Sigma-Aldrich
Iron(III) Chloride, $FeCl_3$			157740-100G	Sigma-Aldrich
cOmplete, Mini EDTA free, protease inhibitor cocktal tablets			4693159001	Roche
10% Mini-Protein TGX Stain-Free Protein Gels			456-8034	Bio-Rad
Trans-Blot Turbo Mini PVDF Transfer Packs			170-4156	Bio-Rad
SuperSignal West Femto Maximum Sensitivity Substrate			34096	ThermoFisher Sci.
Clarity Western ECL Substrate			170-5060	Bio-Rad
Middlebrook 7H9 liquid medium			DF0713-17-9	BD Difco
Middlebrook 7H11 agar			DF0838-17-9	BD Difco
MyTaq DNA polymerase			BIO-21106	Bioline
Aurum Total RNA Mini Kit			732-6820	Bio-Rad
Experion RNA StdSens Analysis Kit			700-7104	Bio-Rad
iScript Adv cDNA Synthesis Kit for RT-qPCR			172-5037	Bio-Rad
SsoAdvanced Universal SYBR Green Supermix			172-5271	Bio-Rad
Phorbol 12-myristate 13-acetate			P8139-10MG	Sigma-Aldrich
Ionomycin calcium salt			10634-5MG	Sigma-Aldrich
Brefeldin A solution (1000X)			00-4506-51	ThermoFisher Sci.

Collagenase-D	11088866001	Sigma-Aldrich
BCA Protein assay kit	23227	ThermoFisher Sci.
Fixation and Permeabilization Solution	554714	BD Biosciences
eBioscience™ Foxp3 / Transcription Factor Staining Buffer Set	00-5523-00	ThermoFisher Sci.
Formalin solution, neutral buffered, 10%	HT501320	Sigma-Aldrich
Mycobacteria selectatab (Kirchner) 1 tab/500mL	MS24	Mast International Ltd
Gibco^{TM} Dulbecco's Modified Eagle Medium (DMEM)	11995065	ThermoFisher Sci.
Gibco^{TM} Dulbecco's Phosphate-Buffered Saline (DPBS)	14040-133	ThermoFisher Sci.
Fetal Bovine Serum	SH3007103	HyClone Fisher Sci.
**Experimental models: organism/strains**	**Identifier**	**Source**
Human subjects (TB patients and healthy controls)	BE019/13	King DinuZulu Hospital
Mycobacterium tuberculosis (H37Rv)	ATCC	
CSE-/- and WT (CSE+/+) mice (C57BL/6J x 129SvEv)	co-author	Rui Wang(1)

Software	Company	Detail
FACS Diva Software	BD biosciences	https://www.bdbiosciences.com/en-us/instruments/research-instruments/research-software/flow-cytometry-acquisition/facsdiva-software
FlowJo 10	FlowJo	https://www.flowjo.com/solutions/ flowjo/downloads
NDPI Tools		https://www.imnc.in2p3.fr/pagesperso/deroulers/software/ndpitools/
Wave Desktop	Agilent	Version 2.6
CFX Manager	Bio-Rad	version 3.1
GraphPad Prism 7	Graphpad Prism	Version 7.04
CorelDRAW	Corel	Version X8
ImageJ	NIH	https://imagej.nih.gov/ij/download.html
pheatmap: Pretty Heatmaps(7)		R package version 1.0.12 (https://CRAN.R-project.org/package=pheatmap)
R: A language & statistical computing		R Foundation, Vienna, Austria (https://www.R-project.org/)
KEGG(8)		https://www.genome.jp/kegg/pathway.html
Mouse Genome Database(9)		http://www.informatics.jax.org/

Primers(1)	Nucleotide sequence
N1	5’-TGCGAGGCCAGAGGCCAGTTGTGTAGC-3’
F1	5’-TGTTCATGGTAGGTTTGGCC-3’

R1	5'-TCAGAACTCGCAGGGTAGAA-3'
CSEqF	5'-GGGCCAGTCCTCGGGTTTTGAATA-3'
CSEqR	5'-TAATCGTAATGGTGGCAG CAAGAC-3'

References

1. Yang G, *et al.* (2008) H_2S as a physiologic vasorelaxant: hypertension in mice with deletion of cystathionine gamma-lyase. *Science* 322(5901):587-590.
2. Cumming BM, Addicott KW, Adamson JH, & Steyn AJ (2018) *Mycobacterium tuberculosis* induces decelerated bioenergetic metabolism in human macrophages. *eLife* 7:e39169.
3. Sugahara S, *et al.* (2016) Colorimetric Determination of Sulfide in Microsamples. *Anal Sci* 32(10):1129-1131.
4. Ewels P, Magnusson M, Lundin S, & Kaller M (2016) MultiQC: summarize analysis results for multiple tools and samples in a single report. *Bioinformatics* 32(19):3047-3048.
5. Dobin A, *et al.* (2013) STAR: ultrafast universal RNA-seq aligner. *Bioinformatics* 29(1):15-21.
6. Love MI, Huber W, & Anders S (2014) Moderated estimation of fold change and dispersion for RNA-seq data with DESeq2. *Genome biology* 15(12):550.
7. Kolde R (2019) pheatmap: Pretty Heatmaps. R package version 1.0.12. *https://CRAN.R-project.org/package=pheatmap*.
8. Kanehisa M & Goto S (2000) KEGG: kyoto encyclopedia of genes and genomes. *Nucleic Acids Res* 28(1):27-30.
9. Bult CJ, *et al.* (2019) Mouse Genome Database (MGD) 2019. *Nucleic Acids Res* 47(D1):D801-D806.
